# Five Years of Experimental Warming Increases the Biodiversity and Productivity of Phytoplankton

**DOI:** 10.1371/journal.pbio.1002324

**Published:** 2015-12-17

**Authors:** Gabriel Yvon-Durocher, Andrew P. Allen, Maria Cellamare, Matteo Dossena, Kevin J. Gaston, Maria Leitao, José M. Montoya, Daniel C. Reuman, Guy Woodward, Mark Trimmer

**Affiliations:** 1 Environment and Sustainability Institute, University of Exeter, Penryn, Cornwall, United Kingdom; 2 School of Biological and Chemical Sciences, Queen Mary University of London, London, United Kingdom; 3 Department of Biological Sciences, Macquarie University, Sydney, Australia; 4 Bi-Eau, Angers, France; 5 Ecological Networks and Global Change Group, CNRS Station Experimentale du Moulis, Moulis, France; 6 CREAF, Centre for Ecological Research and Forestry Applications, Cerdanyola del Vallès, Spain; 7 Department of Ecology and Evolutionary Biology and Kansas Biological Survey, University of Kansas, Lawrence, Kansas, United States of America; 8 Laboratory of Populations, Rockefeller University, New York, New York, United States of America; 9 Department of Life Sciences, Imperial College London, Ascot, Berkshire, United Kingdom; Princeton University, UNITED STATES

## Abstract

Phytoplankton are key components of aquatic ecosystems, fixing CO_2_ from the atmosphere through photosynthesis and supporting secondary production, yet relatively little is known about how future global warming might alter their biodiversity and associated ecosystem functioning. Here, we explore how the structure, function, and biodiversity of a planktonic metacommunity was altered after five years of experimental warming. Our outdoor mesocosm experiment was open to natural dispersal from the regional species pool, allowing us to explore the effects of experimental warming in the context of metacommunity dynamics. Warming of 4°C led to a 67% increase in the species richness of the phytoplankton, more evenly-distributed abundance, and higher rates of gross primary productivity. Warming elevated productivity indirectly, by increasing the biodiversity and biomass of the local phytoplankton communities. Warming also systematically shifted the taxonomic and functional trait composition of the phytoplankton, favoring large, colonial, inedible phytoplankton taxa, suggesting stronger top-down control, mediated by zooplankton grazing played an important role. Overall, our findings suggest that temperature can modulate species coexistence, and through such mechanisms, global warming could, in some cases, increase the species richness and productivity of phytoplankton communities.

## Abstract 2

A five-year mesocosm experiment shows that warmer water temperatures increase the biodiversity and productivity of phytoplankton communities.

## Author Summary

At the global scale, phytoplankton take up about as much carbon dioxide (CO_2_) as the tropical rainforests. However, in spite of their importance in global carbon cycles, we understand very little about how phytoplankton communities and the critical functions they mediate, including CO_2_ sequestration, are likely to change as the climate warms in the coming decades. In this study, we report the results of a five-year warming study in experimental outdoor ponds, known as mesocosms. Warmed (+4°C) communities had 67% more species and higher rates of gross primary productivity (CO_2_ fixation). Our results show that warming resulted in higher productivity by increasing the biodiversity and biomass of the phytoplankton. Warming also changed the species composition of the phytoplankton communities by favouring larger organisms that were more resistant to grazing from zooplankton. Our work demonstrates that future global warming is likely to have major impacts on the composition, biodiversity, and functioning of planktonic ecosystems by affecting metabolic rates and species interactions. The increases in the biodiversity and productivity of the phytoplankton seen in this study also highlights that the effects of a warming environment might not always be adverse for all ecosystems.

## Introduction

Phytoplankton fix CO_2_ from the atmosphere through photosynthesis and underpin the secondary production of many of the world’s aquatic ecosystems [1], yet the effects of global warming on their biodiversity (the number of distinct taxa in a given location) and productivity remains largely unknown. Temperature sets the pace of metabolism [2] and, consequently, a host of life history traits and attributes that determine fitness, including population growth rate, abundance, resource acquisition rate, mortality, and interspecific interactions [3–7]. Global warming could therefore substantially alter local phytoplankton biodiversity through its effects on ecological dynamics (e.g., competition, predation). For example, it could reduce local biodiversity by increasing metabolic rates (and hence resource demands) of individuals, resulting in competitive exclusion through increased resource competition. Alternatively, it could increase local biodiversity by magnifying effects of keystone- or frequency-dependent predation (e.g., where consumers switch between resources preferentially to predate upon the most abundant taxa), allowing inferior competitors to persist [8–10].

Temperature-mediated effects on species coexistence have received relatively little attention and remain poorly understood (but see [9] for a notable exception). While the experiments conducted thus far suggest that phytoplankton biodiversity declines with increases in temperature [11–15], extrapolating to the field is challenging because these primarily laboratory-based experiments are conducted over relatively short time scales (e.g., weeks to months). Such experiments also cannot replicate several key ecological processes that influence local biodiversity dynamics in nature. For example, species turnover occurs in communities through ceaseless immigration and local extinction, the balance of which determines local biodiversity levels [16,17]. Laboratory experiments are disconnected from a regional species pool and exclude dynamics of dispersal-mediated community assembly, which are important for determining metacommunity structure [16]. In addition, such experiments cannot fully capture the material recycling processes that shape natural ecosystem succession [18]. The few experiments conducted in outdoor mesocosms, and which can account for metacommunity dynamics, have not investigated the effects of warming on patterns of biodiversity and species coexistence [19,20].

In contrast to laboratory experiments, eco-evolutionary species distribution models of the responses of phytoplankton biodiversity to ocean warming suggest net losses in the tropics and gains at the poles [21]. These models, however, ignore trophic interactions [21], which could play a central role in determining how warming affects competition and coexistence in phytoplankton communities [22]. These limitations, both in published experiments and in current theory, highlight important gaps in our understanding of how the biodiversity and productivity of phytoplankton communities might respond to global warming.

We used a long-term, outdoor mesocosm experiment to investigate the effects of warming on the structure, biodiversity, and functioning of plankton communities. Our experimental approach differs in several fundamental ways from previous studies that have investigated the effects of warming on phytoplankton biodiversity [11–15]. First, our outdoor mesocosms are open to natural dispersal (aerial and/or transportation on mobile vertebrates) from the regional species pool, allowing us to explore the effects of experimental warming in the context of metacommunity dynamics. Second, because these mesocosms comprise both benthic and pelagic communities, they include fundamental material recycling processes and can recreate many of the salient biogeochemical features of natural aquatic ecosystems [23–26]. Finally, the mesocosms had been warmed for 5 yr at the time of sampling, allowing us to characterize biodiversity responses in the context of long-term successional and ecosystem dynamics over tens-of-thousands of generations for the phytoplankton.

We carried out a detailed empirical survey to assess how long-term experimental warming altered the structure, diversity, and taxonomic composition of the phytoplankton communities. We then characterized how the biomass, size structure, and composition of the zooplankton (the consumers of the phytoplankton) were affected and determined the extent to which warming altered the functioning (e.g., gross primary production [GPP] and community respiration [CR]) of the plankton communities. Finally, we use these measurements, taken across multiple levels of organization (population, community, & ecosystem) to explore the mechanisms that link the structure and dynamics of the plankton community to the ecosystem processes they mediate and the way in which warming altered the functioning of these ecosystems.

## Results

### Phytoplankton Diversity and Community Structure

**Fig 1:**
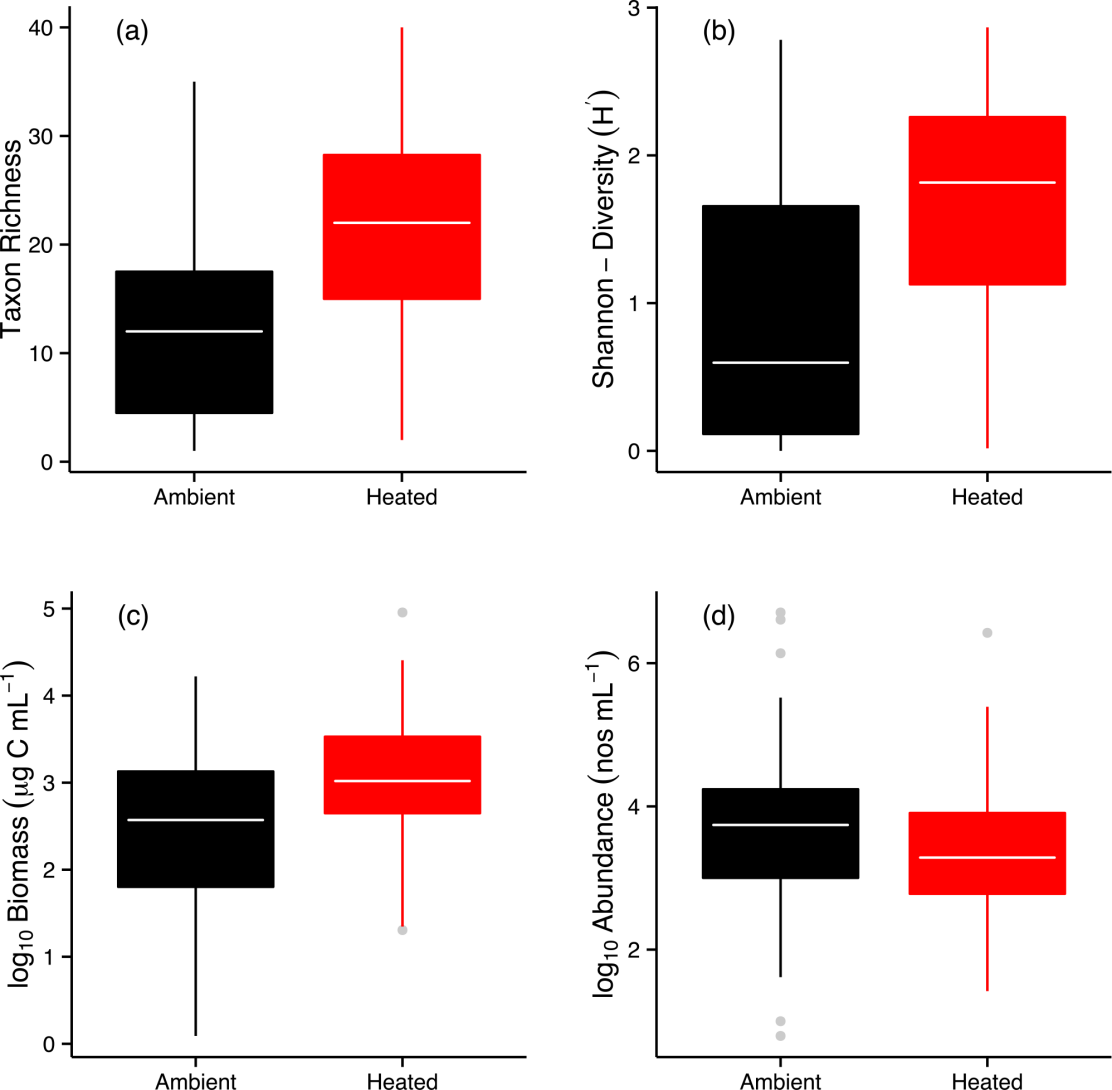
Effects of warming on phytoplankton biodiversity. Analyses reveal that (**a**) local taxon richness, (**b**) the Shannon-Diversity Index, and (**c**) total biomass were significantly elevated on average over the year in the warmed treatments. On the contrary, annual levels of (**d**) total abundance did not differ significantly between treatments (see also S1 Table). Different colors are used to represent warmed (red) and ambient (black) treatments. Tops and bottoms of boxes in box-whisker plots correspond to the 25th and 75th percentiles, horizontal white lines correspond to medians, and whisker extents correspond to 1.5 x the interquartile range. The data underlying these analyses can be found in S1 Data.

Average taxonomic richness of the phytoplankton communities was 67% higher in the warmed mesocosms (Fig 1A; S1 Table). The Shannon Diversity Index also increased with warming (Fig 1B; S1 Table), as did rarefied richness (S3 Fig) and total biomass (Fig 1C; S1 Table), while total abundance was unaffected by warming (Fig 1D; S1 Table). These results demonstrate that the higher diversity of the warmed communities cannot be attributed to greater numbers of individuals.

**Fig 2:**
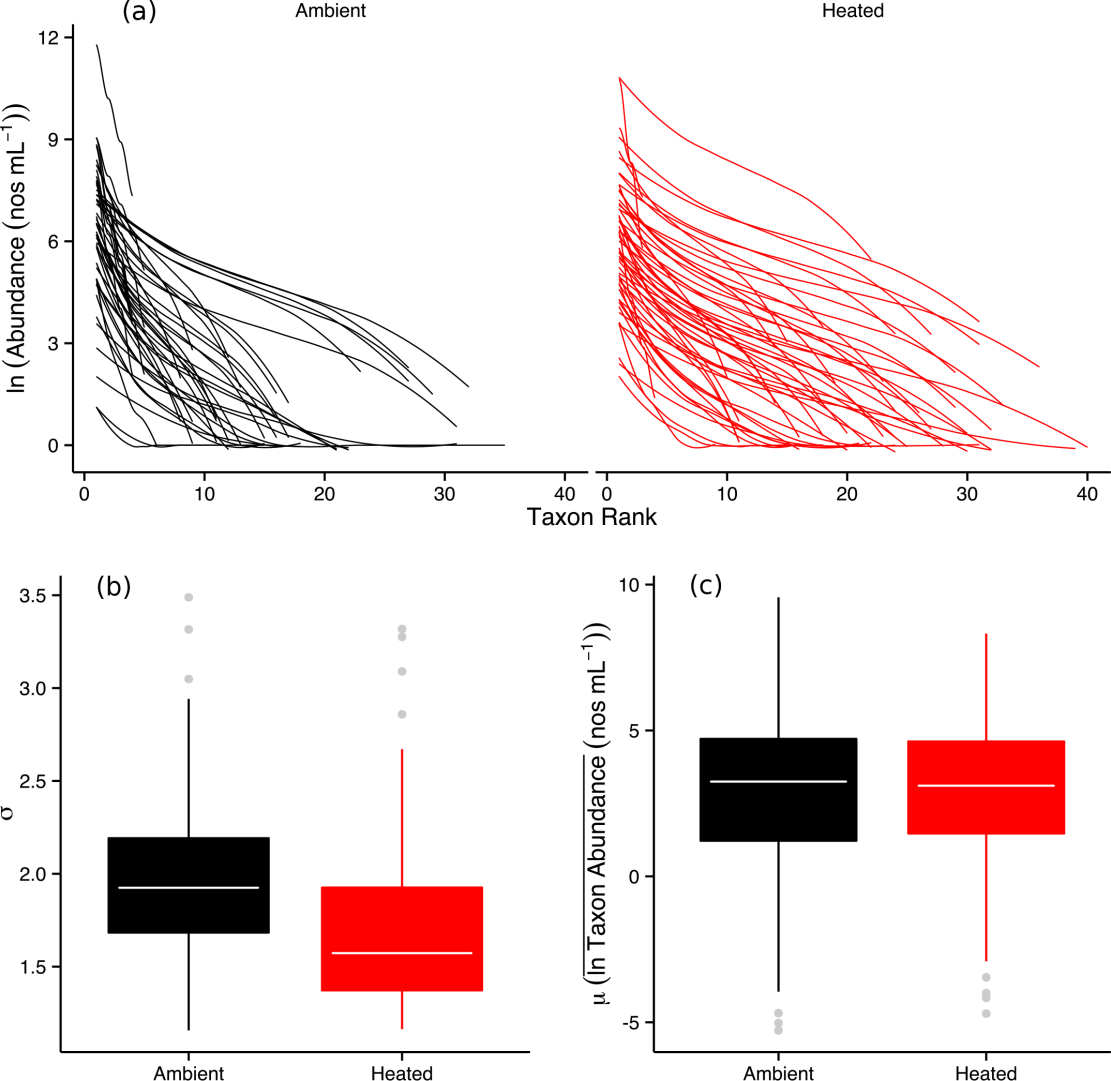
Effects of warming on the species abundance distributions of the phytoplankton communities. (**a**) Rank-abundance curves for each pond on each of the seven sampling occasions for the ambient (black) and heated (red) treatments. Fitted lines represent maximum likelihood fits of the Poisson-lognormal distribution, the parameters of which are given in (**b)** standard deviation of abundance, σ and (**c)** mean log-abundance, μ. The parameter estimates of σ were significantly lower in the warmed treatments (see S1 Table), though estimates of μ were not significantly different between warmed and ambient treatments (see S1 Table). Tops and bottoms of boxes in box-whisker plots correspond to the 25th and 75th percentiles, horizontal white lines correspond to medians, and whisker extents correspond to 1.5 x the interquartile range. The data underlying these analyses can be found in S1 Data.

The rank abundance distributions (RADs) of the phytoplankton communities for the warmed treatments were markedly flatter, with more equitable allocations of individuals among more taxa, compared to their ambient counterparts (Fig 2A). Parameter estimates from the Poisson log-normal fits (see Methods) also demonstrated that the standard deviation of log-abundance per taxon (σ) was lowest in the warmed treatments (Fig 2B; S1 Table), whereas the average of log-abundance per taxon (μ) did not differ between treatments (Fig 2C; S1 Table). Overall, these findings indicate that long-term warming altered patterns of dominance and diversity in these local communities.

**Fig 3:**
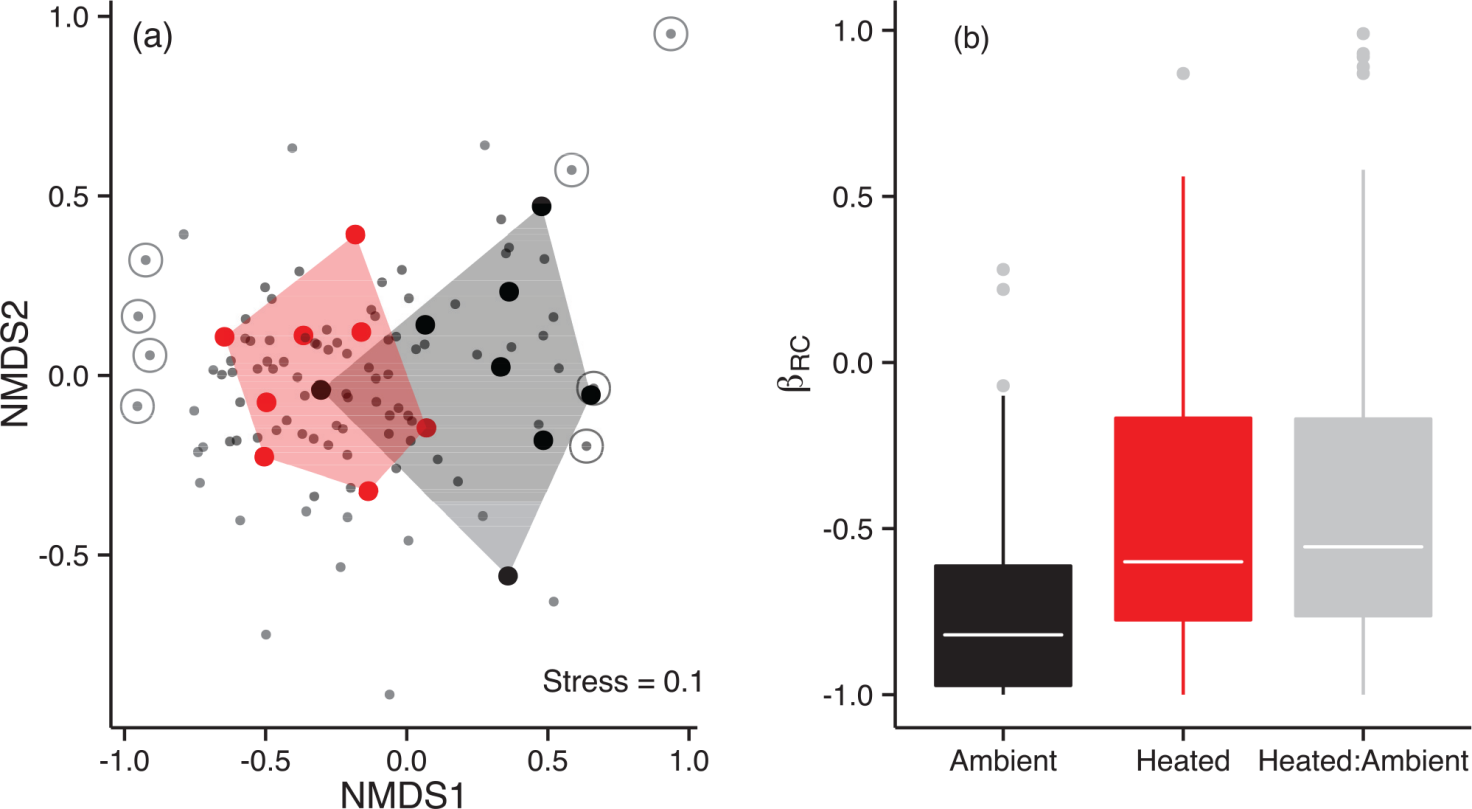
Effects of warming on the taxonomic structure of the phytoplankton. (**a**) NMDS ordination of the phytoplankton communities for each mesocosm. Polygons outline the warmed and ambient treatments, where red circles represent the NMDS scores for each warmed mesocosm, while black circles correspond to the ambient mesocosms. The four taxa most strongly associated with each of the treatments (Warmed: *Anabaena constricta*, *Closterium venus*, *Spirogyra* sp., *Chlamydocapsa* cf., Ambient: *Chromulina* sp., *Tetraedron caudatum*, *Chlorella* spp., *Chlamydomonas* sp.) are highlighted by the grey rings. The ordination demonstrates a clear separation between the warmed and ambient treatments. (**b**) Box whisker plot of rescaled Raup-Crick metrics (β_RC_), which vary from −1 (communities more similar than expected by chance) to 1 (communities more dissimilar than expected by chance) and quantify the relative roles of stochastic and deterministic factors in driving community assembly. β_RC_ values were predominantly < 0 for pairwise comparisons among ambient ponds (black), warmed ponds (red), and between ambient:warmed (grey) contrasts, suggesting deterministic mechanisms played a dominant role in community assembly across the experiment. Tops and bottoms of boxes in box-whisker plots correspond to the 25th and 75th percentiles, horizontal white lines correspond to medians, and whisker extents correspond to 1.5 x the interquartile range. The data underlying these analyses can be found in S1 Data.

Warming also altered the taxonomic composition of the phytoplankton. Nonmetric multidimensional scaling (NMDS) revealed a statistically significant separation in the taxonomic composition of the warmed and ambient treatments (Fig 3A; PERMANOVA; *F*_1,15_ = 3.75; *p* = 0.0019). These patterns could reflect deterministic [27,28] effects of temperature on mechanisms of community assembly, for example by altering optimal thermal niches or by changing species interactions. They could also simply reflect stochastic processes of colonisation and extinction [27,28], or a combination of both stochastic and deterministic factors [29]. To investigate the effects of warming on mechanisms of community assembly, we used a null-model approach [27,28] to ask: do patterns of β-diversity (i.e., pond-to-pond differences in taxonomic composition) deviate from those expected under a completely stochastic assembly process given the observed patterns of α- and γ-diversity (i.e., the “local” (one pond) and “regional” (all ponds) numbers of taxa, respectively)? We used the rescaled Raup-Crick metric (β_RC_), which varies from −1 (communities more similar than expected by chance) to 1 (communities more dissimilar than expected by chance) to quantify the relative roles of stochastic versus deterministic factors in driving metacommunity assembly in our experiment [28]. Values of β_RC_ between ambient (*z* = −2.47, *p* = 0.019), warmed (*z* = −2.44, *p* = 0.013) and ambient-warmed (*z* = −5.86, *p* = 0.0009) comparisons were significantly lower (e.g., more similar) than expected by chance alone (Fig 3B), suggesting that deterministic factors dominated mechanisms of community assembly across the experiment.

**Fig 4:**
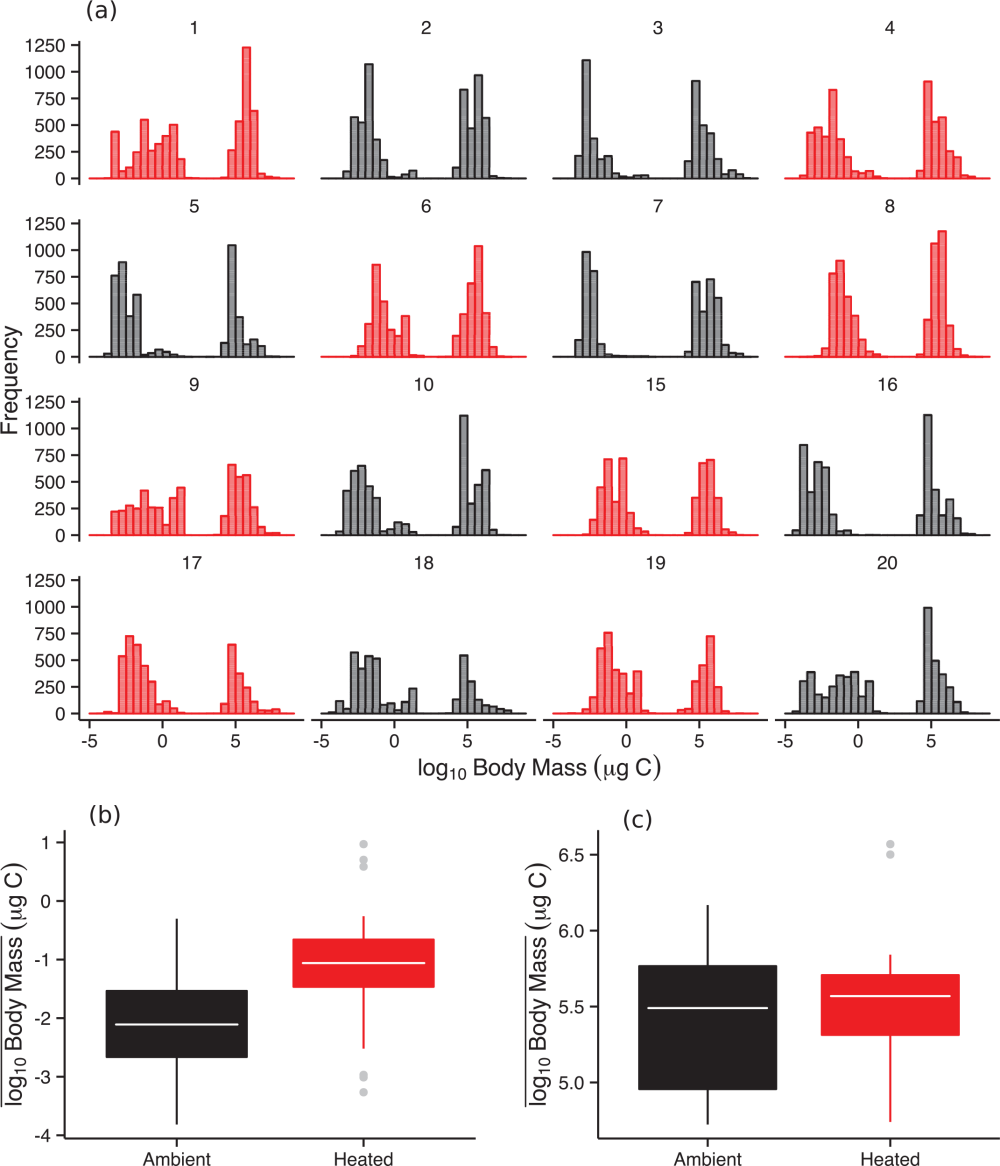
Effects of warming on the body mass distributions of the phytoplankton and zooplankton. (**a**) Frequency distributions of (based on counts) body mass in the plankton communities in each of the 16 mesocosms pooled over the seven sampling occasions (numbers correspond to mesocosm IDs). (**b**) The mean log_10_ body mass of the phytoplankton was significantly higher in the warmed treatments (S1 Table). (**c**) In contrast, mean log_10_ body mass of the zooplankton did not differ between treatments (S1 Table). Tops and bottoms of boxes in box-whisker plots correspond to the 25th and 75th percentiles, horizontal white lines correspond to medians, and whisker extents correspond to 1.5 x the interquartile range. The data underlying these analyses can be found in S1 Data.

The trait distributions of the phytoplankton communities also differed systematically between the warmed and ambient treatments. The latter were dominated by smaller, single-celled genera of algae—e.g., *Chlamydomonas*, *Chlorella*, *Chromulina*—while the former included larger, filamentous cyanobacteria and colonial algae—e.g., *Anabaena*, *Spirogyra*, and *Chlamydocapsa*. Average body masses were an order-of-magnitude higher in the warmed treatments (Fig 4B; S1 Table). Larger phytoplankton taxa, with diameters > 35 μm, are generally considered less susceptible to predation, because they are too large to be handled effectively and consumed by zooplankton [30,31]. Correspondingly, the proportional abundances of these large, inedible phytoplankton taxa were significantly higher in the warmed treatments (S4 Fig; S1 Table).

### Zooplankton Community Structure

**Fig 5:**
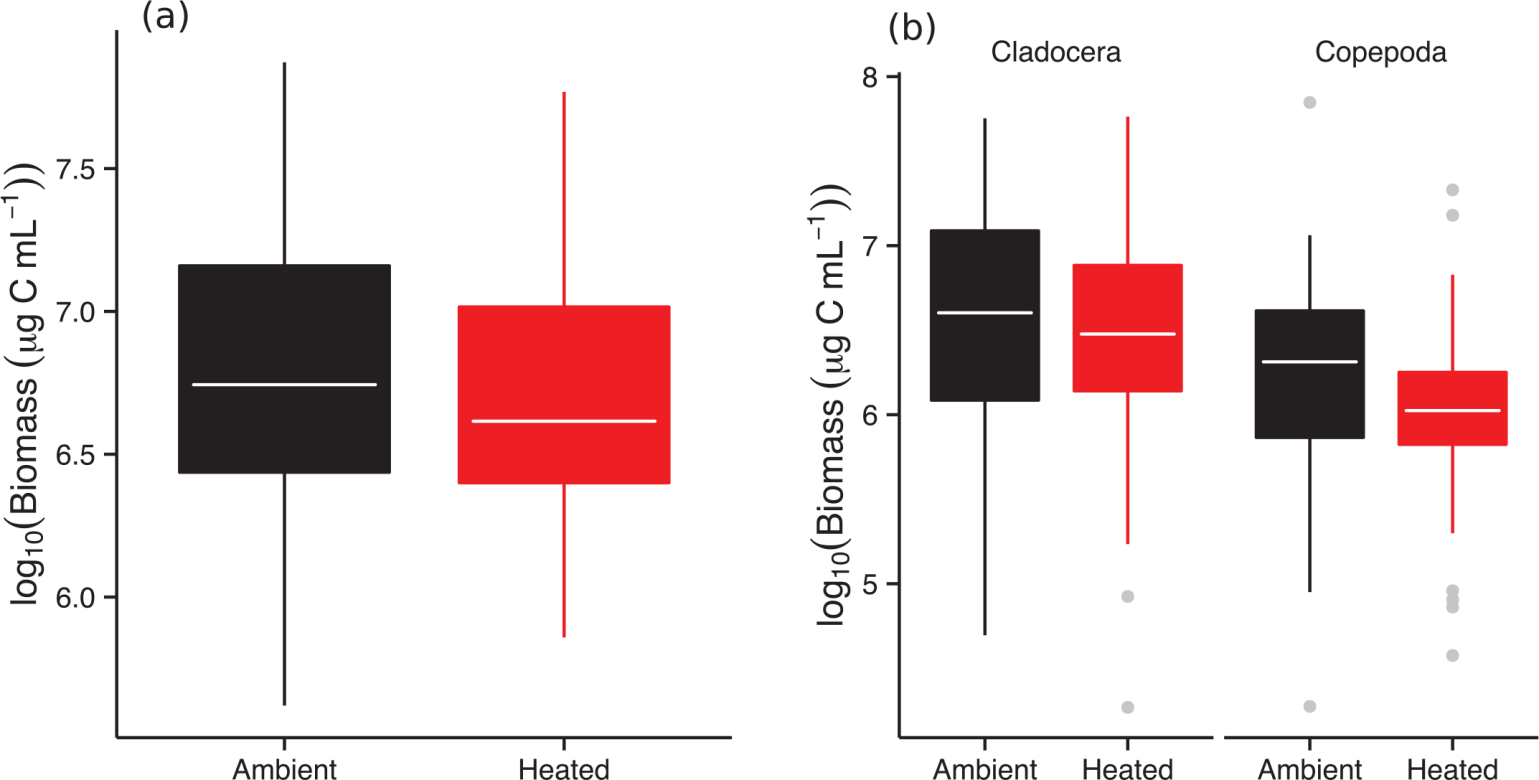
Effects of warming on the biomass and body mass of zooplankton. (**a**) Total biomass of the zooplankton communities, estimated for each mesocosm, on each of the seven sampling months, was not significantly different between the warmed and ambient treatments (S1 Table). (**b**) Consistent with that observed in the whole community, biomass estimates of cladocerans and copepods were also statistically indistinguishable between treatments (S1 Table). Tops and bottoms of boxes in box-whisker plots correspond to the 25th and 75th percentiles, horizontal white lines correspond to medians, and whisker extents correspond to 1.5 x the interquartile range. The data underlying these analyses can be found in S1 Data.

The effects of warming on zooplankton community structure were far weaker than for the phytoplankton: neither total biomass nor average body mass differed significantly between warmed and ambient treatments (S1 Table; Figs 4C and 5A). The total biomass of cladocerans and copepods also did not differ between treatments (S1 Table; Fig 5B). However, further dividing the grazers into the dominant genera revealed some treatment effects, although these were subtle, and apparently compensatory: declines in the biomass of *Diaptomus* were partially compensated by increases in the biomass of *Chydorus* in the warmed treatments, while the biomass of *Daphnia*, *Bosmina*, *Alona*, and ostracods were not affected by warming (S5 Fig).

### Ecosystem Functioning

**Fig 6:**
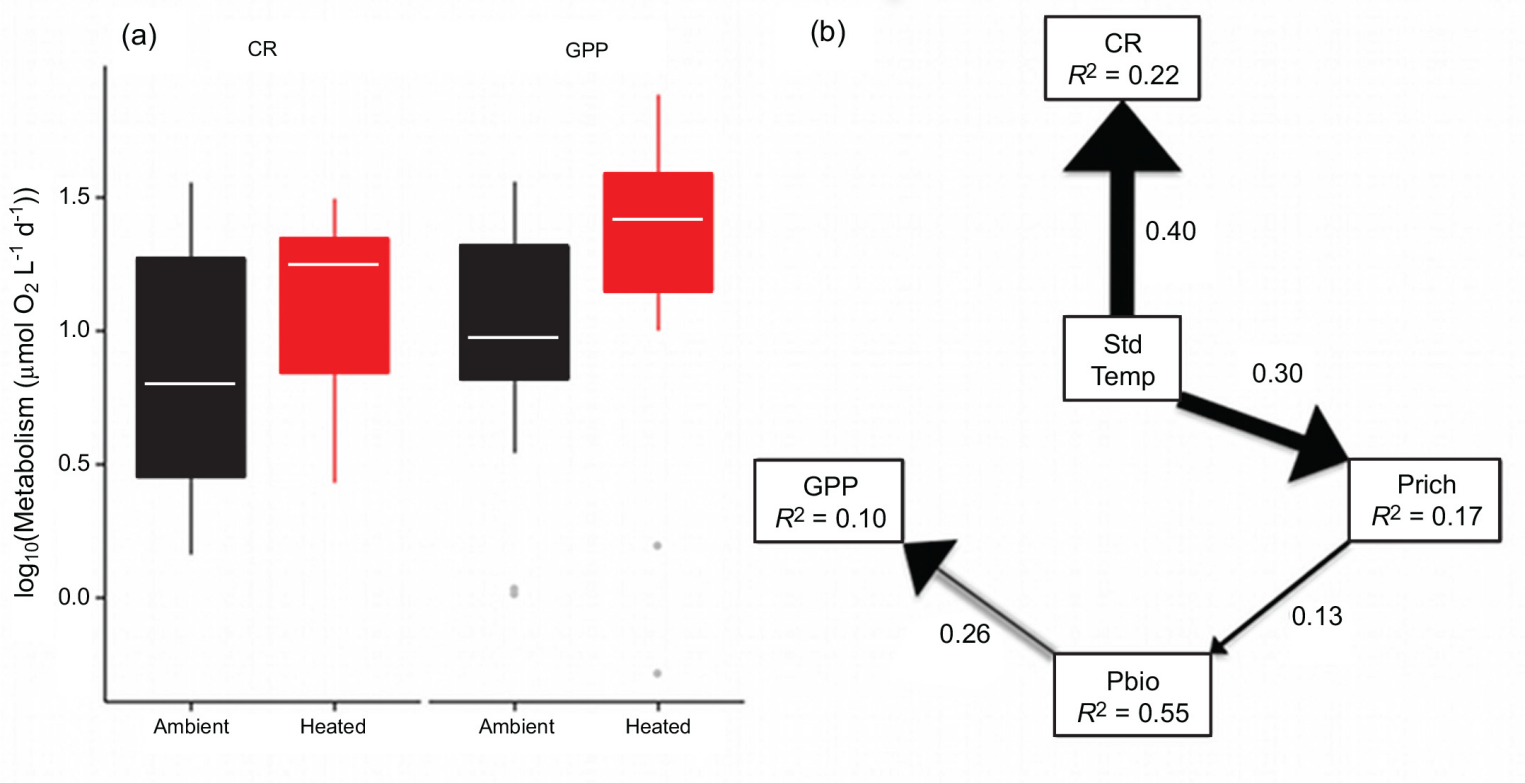
Effects of temperature and plankton community structure on ecosystem functioning. (**a**) Box whisker plot of rates of CR and GPP by treatment, where tops and bottoms of boxes correspond to the 25th and 75th percentiles, horizontal white lines correspond to medians, and whisker extents correspond to 1.5 x the interquartile range. Analyses demonstrate that rates of CR did not differ between treatments, while rates of GPP were significantly elevated (see S1 Table). (**b**) Results of the best-fitting path model (see S2 Table for model selection). Arrow widths are proportional to standardized path coefficients, which are given adjacent to the arrows. Path coefficients are standardized on the observed range and represent the percentage change in the range of the response as the predictor increases across its range. R^2^ values are displayed below endogenous variables (i.e., variables with paths leading to them). Variable codes are as follows: Std Temp (standardized temperature), Prich (phytoplankton taxon richness), Pbio (phytoplankton biomass), GPP, CR, Zbio (zooplankton biomass). The data underlying these analyses can be found in S1 Data.

Gross primary production (GPP) was higher in the warmed treatments, while rates of plankton CR did not differ significantly between treatments (Fig 6A; S1 Table). We used path analysis to test hypotheses about the direct and indirect interactions between warming, the structure of the phytoplankton and zooplankton communities and rates of ecosystem metabolism. In the best fitting model, CR was directly and positively correlated with temperature (Fig 6B). In contrast, warming increased GPP indirectly, because higher temperatures enhanced phytoplankton taxon richness, which in turn elevated levels of phytoplankton biomass and thus rates of GPP (Fig 6B). The best-fitting model also included positive direct effects of temperature on phytoplankton taxon richness (Fig 6). These findings demonstrate that warming altered ecosystem functioning both by directly enhancing metabolic rates (CR) and by indirectly changing the structure of the phytoplankton communities (impacting GPP).

## Discussion

Warming resulted in profound shifts in the organization and biodiversity of the local phytoplankton communities, increasing taxonomic richness by 67% (Fig 1). The distributions of abundance among taxa also became more even (Fig 2), and the taxonomic compositions and distributions of traits (e.g., body mass, edibility) were also altered markedly by warming (Fig 3 & Fig 4). Rates of GPP and CR were elevated in the warmed treatments and path analysis revealed that warming both directly (by stimulating metabolic rates) and indirectly (by increasing the biodiversity and biomass of the phytoplankton) influenced ecosystem functioning. These results demonstrate that ecological mechanisms that determine the number of species that can coexist locally could play an important role in mediating the effects of global warming on the biodiversity and functioning of primary producers in planktonic ecosystems.

Previous experiments have demonstrated that warming tends to reduce the mean body size, total biomass, and biodiversity of phytoplankton communities [14,32–34]. These findings have been attributed to greater competition among phytoplankton for limiting nutrients as a consequence of temperature-induced increases in rates of metabolism and resource uptake [33]. Small species, with high surface area-to-volume ratios and rapid growth rates, are at a selective advantage at high temperatures and low nutrient concentrations and consequently tend to dominate phytoplankton communities under these conditions [1]. Reductions in mean body size at the community level have been coined the “third universal response to global warming” [35,36]. Our results reveal the exact opposite pattern. Phytoplankton communities in the warmed treatments were more species rich, had greater evenness and standing stocks of biomass, and were dominated by larger species. So what mechanism(s) might explain these unexpected findings?

Experimental warming of aquatic food webs tends to enhance top-down regulation of adjacent trophic levels [37–41] by increasing temperature-dependent consumption rates [5–7,42]. High consumption rates can enhance species coexistence when they are frequency-dependent [9,10] and such active prey-switching to target the most abundant resource has been demonstrated for zooplankton [43,44]. It is widely thought to be a key mechanism leading to stable coexistence among resource taxa when strategies leading to higher competitive ability also increase vulnerability to consumption by selective consumers [45–47]. Warming systematically shifted the taxonomic composition of the phytoplankton towards taxa that are more resistant to grazing (Fig 4 & S4 Fig), owing to larger cell size and/or colonial or filamentous growth form [30,48], suggesting stronger top-down control in the warmed treatments. The strength of interactions between consumers and resources increase as a function of the ratio in their body sizes [49] and the ambient temperature [7]. Recent theory predicts that as community assembly dynamics approach immigration–extinction equilibria, consumer–resource body size ratios should converge towards those that are most stable [50]. Our experimental evidence is consistent with this expectation, because increases in the prevalence of large phytoplankton serve to decrease consumer-resource body size ratios (because zooplankton body size was unaffected by warming, see Fig 4C), thereby adding weak trophic interactions to the warmed food webs and counteracting the effects of temperature on interaction strengths by stimulating metabolism.

Warming did not affect the composition, body mass, or biomass structure of the zooplankton. This seems surprising given the major changes observed in the phytoplankton communities. Warming shifted the phytoplankton communities towards large taxa, presumably as a response to selection for grazer-resistant morphology [30,48] owing to higher temperature-dependent consumption rates. The lack of a concomitant shift in zooplankton composition and/or size structure might simply reflect the absence of appropriate traits within the regional species pool. *Daphnia* spp. are among the largest zooplankton grazers in freshwater ecosystems and were the dominant taxon in both the ambient and warmed treatments (S5 Fig). It seems likely that larger taxa, which could track the order-of-magnitude increase in mean body size observed in the phytoplankton, were simply absent from the regional species pool. Nevertheless, the higher temperatures will have increased the metabolic demands of the zooplankton in the absence of any shifts in composition, body mass and biomass structure.

Path analysis demonstrated that plankton CR was directly and positively correlated with temperature. By contrast, warming increased GPP indirectly, because higher temperatures enhanced phytoplankton taxon richness, which in turn elevated levels of phytoplankton biomass and thus rates of GPP (Fig 6B). This result suggests that after controlling for other correlated variables, heterotrophic metabolism (a proportion of CR) was more strongly affected by temperature than rates of autotrophic metabolism (GPP), in line with previous findings in both freshwater and marine ecosystems [25,37,51,52]. Thus, if elevated metabolic rates are correlated with higher consumption rates by heterotrophs, then this once again points towards an increase in the strength of top-down control in the warmed treatments.

The results presented here for the long-term effects of warming on the structure and diversity of phytoplankton contrast sharply with those seen during the first year of this experiment [32] and provide further clues as to the mechanisms responsible for the observed patterns. At the onset of the experiment, the planktivorous fish, *Rutilus rutilus* was added to the mesocosms, but due to poor survivorship, they were removed from all mesocosms at the end of the first year of warming (see Materials and Methods). During the first year of warming, in the presence of the planktivore, genus richness, Shannon-Diversity, and mean body mass of the phytoplankton were all significantly reduced in the warmed treatments, while total abundance increased [33,53] (see S8 Fig). The differential response of the phytoplankton communities in the presence and absence of the fish suggest that warming interacts strongly with the trophic cascade in planktonic communities. When fish are present, their effect on zooplankton releases the phytoplankton from strong, warming-induced, top-down control, resulting in increased interspecific competition for nutrients and selection for small but edible organisms, which are good competitors for nutrients [33,53]. Conversely, when fish are absent, warming increases the strength of top-down control of zooplankton on the phytoplankton, which relaxes interspecific competition for nutrients and favors large and inedible taxa, which are inferior competitors for nutrients, and increases diversity.

The overall weight of experimental evidence (e.g., flatter species abundance distributions, shifts in the taxonomic composition and trait distributions of the phytoplankton towards large colonial or filamentous algae, elevated biomass specific rates of respiration) points towards increases in top-down control, leading to suppression of competitive exclusion and greater species coexistence as the most likely explanation for the increased biodiversity and productivity of the phytoplankton under long-term warming. This is supported by a null model analysis, which demonstrated that deterministic processes dominated mechanisms of community assembly in the phytoplankton, and a path analysis of the variables representing the structure and functioning of the plankton communities. However, without directly manipulating the grazers, we cannot isolate this as the only mechanism driving the shifts in community structure and function in the warmed treatments. Irrespective of the ultimate mechanism, however, our findings demonstrate that in open systems, where local extinctions can be counterbalanced by dispersal-mediated immigration from the regional species pool (e.g., metacommunity dynamics), warming could actually lead to increases (as well as decreases) in biodiversity and ecosystem functioning, in contrast to the received wisdom based on laboratory experiments in closed systems [11–15].

Taken together, our results emphasize the fundamental role temperature plays in constraining patterns of species coexistence and dominance in local communities. They also suggest that warming can alter ecosystem functioning indirectly, by shifting community structure and biodiversity, in addition to its well-known direct effects mediated by metabolism [2]. The findings we report are significant because they show that temperature can enhance the diversity of local communities through ecological mechanisms. Moreover, they mirror biodiversity patterns reported for aquatic and terrestrial taxa along broadscale gradients of temperature and latitude [54]. Latitudinal biodiversity gradients are typically attributed to long-term macroevolutionary and/or historical mechanisms [55]; however, our findings, which isolate the effects of temperature while controlling for other variables that may be confounded along latitudinal gradients (e.g., nutrients, productivity, disturbance regime), suggest that temperature may in part influence local biodiversity through its effects on ecological mechanisms of species coexistence. Through such mechanisms, future global warming could, in some cases, actually enhance species richness and primary productivity in phytoplankton communities.

## Materials and Methods

### Experimental Design

The outdoor mesocosm experiment (see S9 Fig) is based at the Freshwater Biological Association’s River Laboratory (2°10`W, 50°13`N) in East Stoke, Dorset, United Kingdom. Twenty artificial ponds, each holding 1 m^3^ of water, were set up to mimic–shallow standing freshwater ecosystems. The pool of species available for initial colonization was standardized at the outset by seeding all of the ponds in December 2005 with a “common garden” inoculum of organisms from surrounding freshwater habitats. Ponds were then left open to natural colonization and dispersal. Populations of an introduced planktivorous fish, *R*. *rutilus*, were maintained at constant densities (two individuals (age 1+) per mesocosm (~12 g C m^−3^)) in all mesocosms until October 2007. *R*. *rutilus* populations were removed in October 2007 by electrofishing due to ongoing poor survival. Experimental warming began in September 2006 [25] and had run for 5 yr (including 4 yr without fish) at of the onset of sampling in July 2011.

Ten of the twenty ponds were warmed 3–5°C above ambient temperature (see S1 Fig), in accordance with the IPCC A1B global warming projections for the next 100 yr for temperate northern hemisphere regions [56]. Mesocosms were warmed by an electronic heating element connected to a thermocouple, which was used to monitor the temperature in a given pair of warmed and ambient mesocosms. Over the 5-yr experiment, temperatures were logged every 5 min, and minor adjustments were made, if required, to ensure that temperature differences between treatments were ~4°C; the mean annual temperature difference between treatments over the year of sampling was 4.4°C ± SE 0.03 (S1 Fig). During the second year of the experiment, the heating elements in two of the warmed ponds malfunctioned. We therefore removed these replicates from the experiment, as well as the ambient replicates to which they were paired. All analyses presented here are on the 16 remaining replicates (8 warmed; 8 ambient).

### Abiotic Variables

Water temperature was, as expected, significantly elevated in the warmed treatments (S1 Table; S1 Fig). By contrast, total dissolved inorganic nitrogen (S2 Fig) and orthophosphate (S2 Fig) were, on average, statistically indistinguishable between the warmed and ambient treatments over the annual cycle (S1 Table). Taken together, these two results show that of the variables we measured, temperature was the principal abiotic variable altered by experimental warming.

### Plankton Sampling

The plankton community from each of the 16 mesocosms was sampled every two months between July 2011 and July 2012 (7 sampling occasions in total). The entire water column from the sediment surface to the water surface was sampled using a 0.8 m-length tube sampler (Volume: 2 L), which was positioned at random in each mesocosm on each sampling date. Each sample was divided into two size categories (>100 μm, <100 μm) for preservation and subsequent analyses, via filtration through a 100 μm aperture sieve: organisms >100 μm were preserved in 4% Formalin, and a 100 mL subsample of organisms <100 μm was preserved in 1% Lugol’s iodine.

Phytoplankton <100 μm were counted using a LEICA DMIRB inverted microscope at 400x magnification, following the Utermöhl method [57]. The microscope was connected to an interactive image analysis system (LEICA EC3 camera and LAS software) to allow for a higher magnification. For each sample, at least 400 individuals (single cell, colony or filament) were counted, measured, and identified. Counts were converted to volumetric estimates of abundance (organisms mL^−1^) based on the volume of sample analyzed, which varied between 1 and 25 mL depending on the density of organisms. In total, 171 taxa were identified, 85% of which were identified to species level; the remaining 15% were identified to genus or class, or were undetermined (see S4 Table).

Plankton >100 μm (typically zooplankton) were counted, measured, and identified using a Nikon SMZ1500 dissection microscope. Of the six most dominant zooplankton taxa, which together accounted for 91% of the total zooplankton biomass, five were identified to genus level and one to class. This group included the key cladoceran and copepod genera (e.g., *Daphnia* spp., *Bosmina* spp., *Chydorus* spp., *Alona* spp., *Diaptomus* spp.) as well as the ostracods, which are important planktonic grazers in lakes [58]. The remaining taxa were identified to the highest possible taxonomic level, typically class or family.

Linear dimensions of each individual were determined using image analysis. The “size” of each organism was expressed in units of carbon mass (μg C). To estimate masses of organisms >100 μm (typically zooplankton), biovolumes were first determined by assigning organisms to geometric shapes that closely represented the real shape of the organism [59]. Masses were then calculated by converting biovolume to fresh weight using a factor of 1.1 g mL^−1^ and converting fresh weight to carbon content assuming a dry:wet weight ratio of 0.25 and a dry carbon content of 40% [59]. To estimate masses of organisms <100 μm (typically phytoplankton), biovolumes were estimated by assigning organisms to matching geometric shapes [60]. For all phytoplankton individuals, biovolume was estimated by considering the linear dimensions of the whole organism, this included single cells (e.g., *Chlorella*), entire filaments (e.g., *Anabaena planctonica*), and colonies along with their mucilage (e.g., *Aphanothece*, *Chlamydocapsa*, *Nephrocytium*, *Sphaerocystis*) [60]. Biovolume was then converted to carbon units following Montagnes et al. [61]. In total, >83,000 organisms, including both phytoplankton and zooplankton, were measured and identified.

### Planktonic and Benthic Metabolism

On four of the seven sampling occasions (May, July, September, November) spanning the main growing season, we measured in situ rates of planktonic and benthic CR and GPP in each mesocosm. At midday, on each of the sampling occasions, we incubated samples of 0.5 L of the pelagic plankton community of each pond in custom-made paired clear and opaque polycarbonate chambers. At the same time, benthic communities were incubated in custom-made paired clear and opaque bottomless chambers that were screwed into the sediment to a depth of approximately 5 cm [62]. Both the pelagic and benthic chambers were equipped with magnetic stirrers (rotating at 300 rpm) to ensure even mixing within the chamber and with a Unisense OX50 microelectrode to measure the net production or consumption of O_2_ over a 30-min incubation period. Net community production (NCP) was measured as the change in O_2_ concentration in the clear chamber, while CR was the change in O_2_ in the opaque chamber. The concentration of O_2_ was measured every minute, and the rate of metabolism (NCP or CR) was determined as the slope of a linear regression between O_2_ (μmol O_2_ L^−1^ – pelagic; μmol O_2_ m^−2^ – benthic) and time (h). GPP (μmol O_2_ L^−1^) was estimated as the sum of the NCP and CR terms for both the pelagic and benthic zones, taking the appropriate chamber volumes and sediment surface areas into account.

### Nutrient Analyses

Water samples for measuring dissolved inorganic nutrient concentrations were collected from mid-depth in each mesocosm at 9:00 a.m. on each sampling occasion. Samples were filtered (Whatmann GF/F) and stored frozen at −20°C for subsequent determination of NO_3_^−^, NO_2_^−^, NH_4_^+^, and orthophosphate (HPO_4_^2−^ + PO_4_^3−^) using a segmented flow auto-analyzer (Skalar, San++, Breda, Netherlands) [63].

### Statistical Analyses

#### Assessing treatment effects on abiotic and biotic variables

The effects of warming on abiotic (temperature, nutrients) and biotic variables (including phytoplankton taxon richness, diversity, abundance and biomass) were assessed using repeated measures ANOVA, fitting treatment (two levels: warmed and ambient) as a fixed effect, and mesocosm (16 levels) and sampling month (7 in total) as random effects on the intercept. All ANOVA models were fitted using the function “lmer” in the “lme4” package for R statistical software [64]. Significance of the effect of warming was assessed using a likelihood ratio test by fitting nested models with and without the treatment variable using the maximum-likelihood method, and then comparing the two models using a likelihood ratio test (details given in S1 Table). Parameter estimates were obtained by refitting models using restricted maximum likelihood, as recommended in Zuur et al. [65]. Response variables (described below) were log_10_ transformed prior to analysis when they did not conform to a normal distribution.

We assessed the effects of warming on the diversity of phytoplankton communities by submitting phytoplankton taxon richness and Shannon diversity to repeated measures ANOVA, as described above, using diversity estimates for each mesocosm on each of the seven sampling occasions. We also compared the RADs of warmed and ambient phytoplankton communities from each of the sampling months. Species-abundance data were fitted to the Poisson log-normal distribution using maximum-likelihood fitting implemented with the “mle2” function in the “bbmle” package in R statistical software [64]. Samples with less than three taxa (*n* = 12) were dropped from the analysis because the data were insufficient for model fitting. We used the two fitted parameters of the Poisson log-normal distribution (μ and σ) as metrics of the average log-taxon abundance and the relative evenness of the distribution of log-abundances among taxa, respectively, and submitted these variables to repeated measures ANOVA to test for the effect of treatment.

Abundance and biomass-related response variables submitted to repeated measures ANOVA included total phytoplankton abundance and biomass, mean phytoplankton body mass, mean zooplankton body mass, and total biomass estimates of the key zooplankton functional groups—e.g., copepods, cladocerans, ostracods—and selected zooplankton genera, which were the main grazers in our experiment (*Daphnia*, *Bosmina*, *Chydorus*, *Alona*, *Diaptomus*).

Effects of warming on phytoplankton community composition were assessed using two metrics. The first—proportional abundance of inedible phytoplankton—was used as a potential indicator of predation pressure [30]. Following Litchman et al. [30], we defined inedible taxa as those with greatest axial linear dimensions (GALDs) > 35 μm, because they are too large to be handled effectively and consumed by zooplankton. The second—a taxonomic composition metric—was indexed as the “score” for each mesocosm along the first axis of a NMDS ordination. NMDS ordination was conducted using the “metaMDS” function in the “vegan” package in R [64] based on a Bray–Curtis dissimilarity matrix derived from log_10_ (*x* + 1)-transformed total abundances of the taxa in each mesocosm pooled across the seven sampling months. NMDS projected this matrix into a new coordinate space with a small number of dimensions (in this case, two) while preserving the original Bray–Curtis dissimilarities among samples to the extent possible. Orthogonal rotation was applied to the axes in this new coordinate space so as to maximize the variance in “scores” among samples along the first NMDS axis. Thus, samples with more similar scores along the first NMDS axis are more similar to each other with respect to the dominant gradient in taxonomic composition. Finally, we used Permutational Multivariate Analysis of Variance (PERMANOVA) to test whether Bray–Curtis dissimilarities between warmed and ambient treatments were significant [66].

To determine the dominant mechanisms of community assembly, we analyzed patterns of β-diversity (e.g., pond-to-pond dissimilarity in taxonomic composition) across the experimental metacommunity. We used a null-model approach to determine the relative influence of stochastic versus deterministic processes in constraining patterns of community assembly [27,28]. Because α-, β-, and γ-diversity covary [27,28], warming may influence β-diversity simply because it increases or decreases the ratio of α- to γ-diversity, which would imply community assembly patterns arise simply through stochastic sampling of regional species pool (defined as the cumulative richness across all ponds and all treatments). Alternatively, if β-diversity is higher or lower than expected by random sampling alone (given the observed patterns of α- and γ-diversity), this would support the importance of deterministic community assembly.

We assessed whether long-term experimental warming influenced the relative importance of deterministic and stochastic processes on community assembly using a recently developed null-model approach [27,28], which has been used in the past to interpret a range of diversity correlates, e.g., productivity [67], keystone predation [68], and disturbance [69]. Applying this approach involved simulating taxonomic assemblages in each mesocosm by randomly sampling from the regional species pool while preserving the taxon richness in each mesocosm (i.e., its α-diversity) and the relative occupancy of taxa across the metacommunity (i.e., the probability of a taxon’s occurrence, was weighted by their relative occupancy in the metacommunity) to generate an estimate of the β-diversity expected based solely on stochastic assembly processes [27,28]. Expected β-diversity for each pairwise combination of ponds on each sampling occasion was then calculated as the mean across 10^4^ iterations of the null model. We then tested whether the observed β-diversity differed significantly from that predicted by the simulations from the null model (i.e., expected β-diversity) for warmed and ambient ponds using the “oecosimu” function in the “vegan” package for R [64]. We rejected the null hypothesis of no difference between observed and expected β-diversity if the observed β-diversity was outside the 95% confidence interval of the simulated values. Finally, we calculated the rescaled Raup-Crick metric (β_RC_) to quantify the relative roles of stochastic and deterministic factors in driving metacommunity assembly in our experiment [28]. A β_RC_ value of zero would indicate a dominant role for stochastic assembly mechanisms, whereas values approaching −1 indicate that communities are more similar than expected by chance, while those close to 1 suggest communities are more dissimilar than expected by chance alone.

### Biotic and Abiotic Controls on Ecosystem Functioning

We used path analysis (a variant of structural equation modeling that uses only observed variables) to determine the extent to which warming altered ecosystem functioning directly, by stimulating rates of metabolism, or indirectly, by also altering the biomass and diversity of the plankton communities. Path analysis is a useful tool for studying complex biological systems, because it allows the user to link together component models for different response variables, enabling estimation of direct and indirect effects as well as the overall fit of a complex causal network of influence [70]. Because our experimental design resulted in hierarchical data, with multiple measurements of variables made seasonally, nested within replicate mesocosms, we used Shipley’s method of building a multilevel path model using directional separation tests [71]. This approach entailed assembling a path model as a set of hierarchical linear mixed effects models, each of which included hypothesized relationships between a response variable and a set of predictors as fixed effects and mesocosm ID as a random effect on the intercept. Each mixed effects model was fitted using the “lme” function in the “nlme” package, and the overall path model combining models for each response variable was fit using the “piecewiseSEM” package in R [72]. The predictors included temperature as an exogenous variable (i.e., whose variance arise outside of the model) and GPP, CR, phytoplankton taxon richness, phytoplankton biomass, and zooplankton biomass as endogenous variables (i.e., those whose variance the model seeks to explain). All variables were natural-log transformed (except for temperature) and standardized using the mean to linearize relationships and reduce correlations between the coefficients.

We hypothesized that warming both directly stimulated rates of GPP and CR by simply increasing metabolic rates and indirectly influenced these fluxes by altering the richness and biomass of the phytoplankton and zooplankton communities. We developed a set of candidate models, starting with a full model, which included all feasible paths between variables (see S7 Fig). All paths between variables were treated as “free parameters”, and their direction and magnitude were estimated from the observed data by the model. We then removed paths to generate a set of candidate models and selected among models with two criteria. First, we used Shipley’s test for directional separation, which combines the significance of unrealized paths into a single Chi-squared distributed Fisher’s C statistic as a measure of goodness of fit [71]. For each candidate model that passed this test of adequate fit (e.g., *p* > 0.05), we computed a small sample-size corrected AIC score (AICc) [73]. We then compared between models by calculating delta AICc values and AIC weights (S2 Table). The relative importance of paths in the final model were compared using standardized coefficients, which are dimensionless and express a percentage change in the observed range of the response as the predictor increases across its range.

## Supporting Information

S1 Data Underlying data used in all analyses and figures. (XLSX)

S1 Fig Seasonal variation in and treatment effects on water column temperature over the study year (2011–2012). Box-whisker plots of monthly variation in mean daily water column temperature (*n* = 4,570) in which the top and bottom of the box show the 25th and 75th percentiles and the white lines give the median. Warmed mesocosms are in red while the ambient are in black. Annual means: ambient = 13.4°C, warmed = 17.7°C. The data underlying this analysis can be found in S1 Data.
(EPS)

S2 Fig Seasonal variation in and treatments effects on dissolved inorganic nutrients. (a) Total dissolved inorganic nitrogen (i.e., the sum of NH_4_^+^, NO_3_^−^, NO_2_^−^) exhibited seasonal variation but did not differ significantly between ambient and warmed treatments (see S1 Table). (b) Dissolved PO_4_^3−^ also varied seasonally but did not differ significantly between ambient and warmed treatments (see S1 Table). Box-whisker plots in which the top and bottom of the box show the 25th and 75th percentiles, and the white lines give the median. The data underlying these analyses can be found in S1 Data.
(EPS)

S3 Fig Correlation between observed and rarefied taxon richness. To test whether slight differences in the numbers of organisms counted among replicates influenced our estimates of taxon richness, we generated rarefied estimates of richness based on fixed counts of 400 individuals. These estimates were essentially identical to our observed estimates of richness (slope ~ 1, intercept ~ 0). The data underlying this analysis can be found in S1 Data.
(EPS)

S4 Fig Proportional abundance of inedible phytoplankton. The proportional abundance of inedible phytoplankton was calculated for each mesocosm on each of the seven sampling occasions as the total abundance of phytoplankton with GALD > 35 μm divided by the total abundance of all individuals in the community. Analyses demonstrate that the proportional abundance of inedible individuals was significantly higher in the warmed treatments (see S1 Table). Tops and bottoms of boxes in box-whisker plots correspond to the 25th and 75th percentiles, and horizontal white lines correspond to medians. The data underlying this analysis can be found in S1 Data.
(EPS)

S5 Fig Effects of warming on the biomass of dominant zooplankton grazers. Together, *Daphnia* spp., *Bosmina* spp., *Chydorus* spp., *Alona* spp., *Diaptomus* spp. and Ostracods accounted for 91% of the total zooplankton biomass pooled across all mesocosms over the year. Warming had subtle effects on the relative contribution of the key grazer genera to overall grazer biomass. Declines in the biomass of *Diaptomus* spp. were compensated for by increases in the biomass of *Chydorus* spp. in the warmed treatments, while the biomass of *Daphnia* spp., *Bosmina* spp., *Alona* spp., and Ostracoda were not affected by warming (S1 Table). Tops and bottoms of boxes in box-whisker plots correspond to the 25th and 75th percentiles, and horizontal white lines correspond to medians. The data underlying this analysis can be found in S1 Data.
(EPS)

S6 Fig Ratio of pelagic to benthic GPP. Both benthic and pelagic GPP were expressed on an areal (m^−2^) basis and the effects of treatment were assessed using a mixed effects model on the log_10_-transformed ratio. Treatment was included as a fixed effect, with mescosm and sampling month as crossed random effects on the intercept. Analyses demonstrate pelagic CR was generally larger than that of the benthos and the ratio of pelagic to benthic CR was not significantly different between treatments (χ^2^ = 2.53; d.f. = 1; *p* = 0.11). Similarly, the ratio of pelagic to benthic CR was also indistinguishable between treatments (χ^2^ = 1.39; d.f. = 1; *p* = 0.24). Box-whisker plots in which the top and bottom of the box show the 25th and 75th percentiles, and the white lines give the median. The data underlying this analysis can be found in S1 Data.
(EPS)

S7 Fig Effects of temperature and plankton community structure on ecosystem functioning. Candidate path models used to test hypotheses about the effects of warming on community structure and function. Titles (F0–F11) correspond to models given in the model selection S2 Table. Greyed out arrows highlight putative paths selected for removal in the model selection procedure. R^2^ values are displayed below endogenous variables (i.e., variables with paths leading to them). Variable codes are as follows: Std Temp (standardized temperature), Prich (phytoplankton taxon richness), Pbio (phytoplankton biomass), GPP, CR, Zbio (zooplankton biomass). The data underlying this analysis can be found in S1 Data.
(EPS)

S8 Fig Effects of temperature and plankton community structure on ecosystem functioning. Candidate path models used to test hypotheses about the effects of warming on community structure and function. Titles (F0–F11) correspond to models given in the model selection S2 Table. Greyed out arrows highlight putative paths selected for removal in the model selection procedure. R^2^ values are displayed below endogenous variables (i.e., variables with paths leading to them). Variable codes are as follows: Std Temp (standardised temperature), Prich (phytoplankton taxon richness), Pbio (phytoplankton biomass), GPP, CR, Zbio (zooplankton biomass). The data underlying this analysis can be found in S1 Data.
(EPS)

S9 Fig Effects of temperature and plankton community structure on ecosystem functioning. Candidate path models used to test hypotheses about the effects of warming on community structure and function. Titles (F0–F11) correspond to models given in the model selection S2 Table. Greyed out arrows highlight putative paths selected for removal in the model selection procedure. R^2^ values are displayed below endogenous variables (i.e., variables with paths leading to them). Variable codes are as follows: Std Temp (standardised temperature), Prich (phytoplankton taxon richness), Pbio (phytoplankton biomass), GPP, CR, Zbio (zooplankton biomass). The data underlying this analysis can be found in S1 Data.
(EPS)

S10 Fig Patterns of phytoplankton community structure in 2007 after 1 yr of warming. In 2007, following 1 yr of warming and when *R*. *rutilus* were present as the top predators, we carried out a more restricted survey of the phytoplankton communities (see [32]). In the 2007 survey, the mesocosms were sampled once in April and once in October, and phytoplankton taxa were counted, measured, and identified to genus level following the same methodology outlined in the present study (see Methods). Here, we reanalyze these data and present them for comparison to the 2011–2012 data in which the mesocosms had been warmed for ~5 yr, and *R*. *rutilus* had been absent for ~4 yr (see Methods). The response variables were analyzed using mixed effects models, with treatment included as a fixed effect and with mescosm and sampling month as random effects on the intercept to control for repeated measures on mesocosms. Box-whisker plots in which the top and bottom of the box show the 25th and 75th percentiles and the white lines give the median are presented. (a) Genus richness (χ^2^ = 4.56; d.f. = 1; *p* = 0.03), (b) Shannon-Diversity (χ^2^ = 10.06; d.f. = 1; *p* = 0.0015), and (c) average body mass (χ^2^ = 7.19; d.f. = 1; *p* = 0.0073), were all significantly lower in the warmed treatments, while (d) total abundance was significantly elevated (χ^2^ = 9.32; d.f. = 1; *p* = 0.0022). The effects of warming on the structure and biodiversity of the phytoplankton communities shown here for 2007 when *R*. *rutilus* were present are directly opposite to those in 2011–2012 following their absence for 4 yr. These results suggest that warming strongly interacts with the trophic cascade in planktonic communities—when fish are present, their effect on zooplankton releases the phytoplankton from strong, warming-induced top-down control, resulting in reduced richness, Shannon-Diversity, and mean body mass, but increased abundance. By contrast, when fish are absent, warming increases the strength of top-down control on the phytoplankton, which relaxes interspecific exploitation competition and increases diversity. The data underlying this analysis can be found in S1 Data.
(EPS)

S11 Fig Photograph of the mesocosms experiment. Warmed mesocosms can be identified as those with the small white boxes attached (heating elements). During the second year of the experiment, the heating elements in two of the warmed ponds malfunctioned. We therefore removed these replicates, and the ambient replicates to which they were paired, from the experiment. All analyses presented here are on the 16 remaining replicates (8 warmed; 8 ambient).
(JPG)

S1 Table Results of repeated measures ANOVAs. Linear mixed effects models were fitted to each of the response variables, treating “treatment” as a fixed categorical variable and “mesocosm” and “sampling month” as random effects on the intercept (accounting for repeated measurements on each replicate across months). The most complex models including “treatment” were fit using maximum likelihood, and its significance was assessed by comparing it against a model with a common intercept across both treatments using likelihood ratio tests. Significant *p*-values are highlighted in bold. The data underlying these analyses can be found in S1 Data.
(DOCX)

S2 Table Model selection on candidate path models. The predictors included temperature (Std Temp) as an exogenous variable (i.e., whose variance arise outside of the model) and GPP, CR, phytoplankton taxon richness (Prich), phytoplankton biomass (Pbio), and zooplankton biomass (Zbio) as endogenous variables (i.e., those whose variance the model seeks to explain). We developed a set of candidate models (see S7, S8 and S9 Figs), starting with a full model (F0), which included all feasible paths between variables. We then removed paths with the lowest *p*-values and compared candidate models using small sample size corrected AICc and a goodness of fit determined from D-separation tests (see Methods). Delta AICc is the difference in AICc score relative to the model with the lowest value (most parsimonious model), and Akaike Information Criterion (AIC) Weight (Wt) is the relative support for the model. Path diagrams for all candidate models are given in S7, S8 and S9 Figs. Note that goodness of fit could not be calculated from the fully saturated model (F0). The data underlying this analysis can be found in S1 Data.
(DOCX)

S3 Table Maximum likelihood statistics for the Poisson log-normal fits. Maximum likelihood fits of the Poisson log-normal (parameters: μ and σ) model to each rank-abundance distribution along with the AIC and *p*-value for each parameter. The data underlying this analysis can be found in S1 Data.
(DOCX)

S4 Table List of phytoplankton taxa observed in the warmed and ambient mesocosms. (DOCX)

S5 Table List of zooplankton taxa observed in the warmed and ambient mesocosms. (DOCX)

S6 Table Average annual concentrations of total inorganic nitrogen and orthophosphate. (DOCX)

## Abbreviations

AIC: Akaike Information Criterion
CR: community respiration
GPP: gross primary production
NMDS: nonmetric multidimensional scaling
RAD: rank abundance distribution
