# Supplementary figures and images for: Five Years of Experimental Warming Increases the Biodiversity and Productivity of Phytoplankton
Source: PLoS Biol. 2015 Dec 17;13(12):e1002324. doi: 10.1371/journal.pbio.1002324 (PMC4682994; doi:10.1371/journal.pbio.1002324)

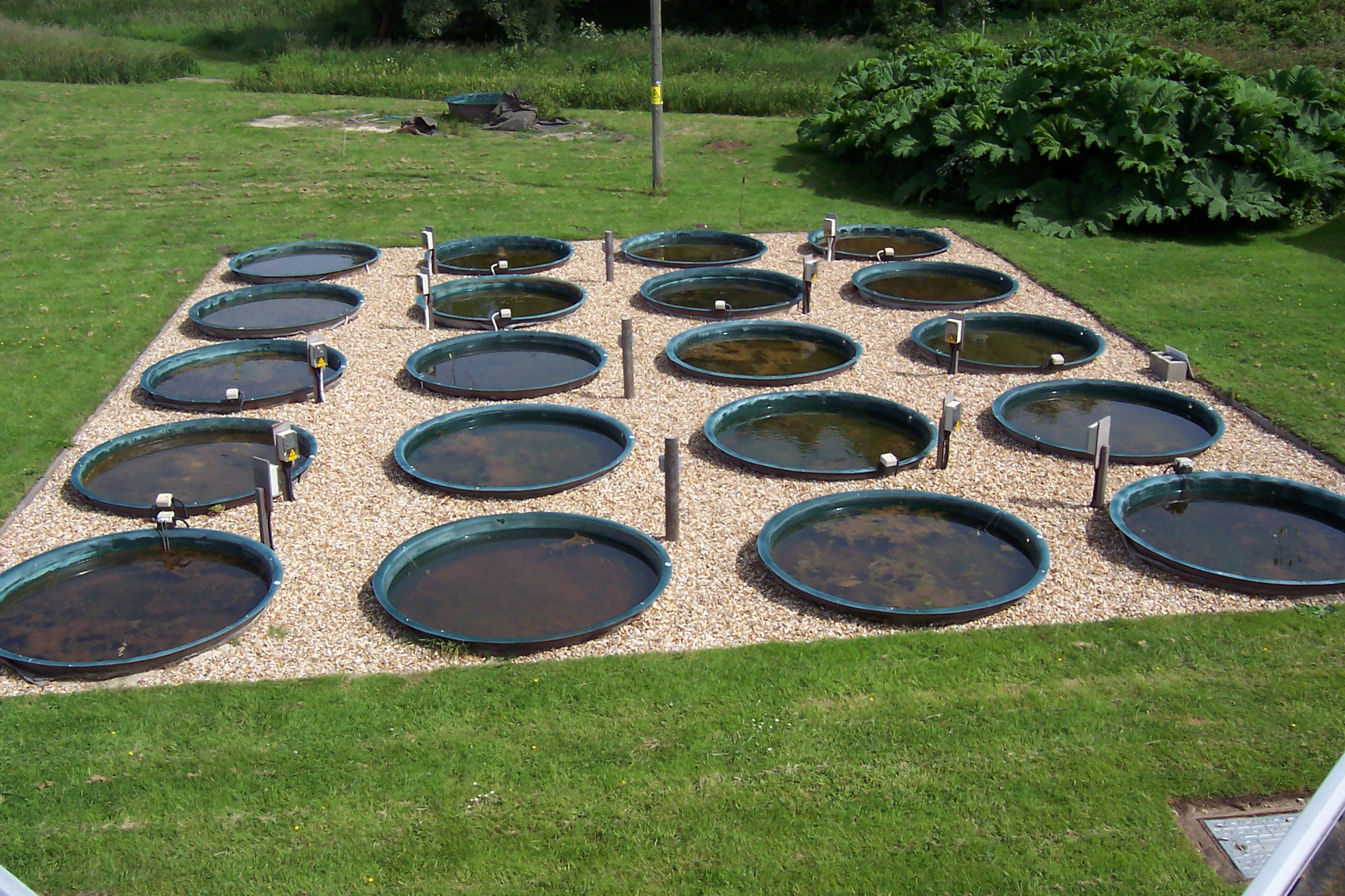

Supplement: S11 Fig — Warmed mesocosms can be identified as those with the small white boxes attached (heating elements). During the second year of the experiment, the heating elements in two of the warmed ponds malfunctioned. We therefore removed these replicates, and the ambient replicates to which they were paired, from the experiment. All analyses presented here are on the 16 remaining replicates (8 warmed; 8 ambient). (JPG) [file pbio.1002324.s012.jpg]
