# Supplementary material for: Five Years of Experimental Warming Increases the Biodiversity and Productivity of Phytoplankton
Source: PLoS Biol. 2015 Dec 17;13(12):e1002324. doi: 10.1371/journal.pbio.1002324 (PMC4682994; doi:10.1371/journal.pbio.1002324)
Supplement: S1 Table — Linear mixed effects models were fitted to each of the response variables, treating “treatment” as a fixed categorical variable and “mesocosm” and “sampling month” as random effects on the intercept (accounting for repeated measurements on each replicate across months). The most complex models including “treatment” were fit using maximum likelihood, and its significance was assessed by comparing it against a model with a common intercept across both treatments using likelihood ratio tests. Significant p-values are highlighted in bold. The data underlying these analyses can be found in S1 Data. (DOCX) [file pbio.1002324.s013.docx]

**S1 Table. Results of repeated measures ANOVAs.**

| ***Fixed Effects*** |  |  |  |  | ***Random Effects*** |  |
| --- | --- | --- | --- | --- | --- | --- |
| **Model** | **Df** | ***n*** | **χ^2^** | ***P*** | **Form** | **Within-group σ** |
| *a) Phyto Taxon Richness* |  |  |  |  | 1\|mesocosm | 4.700 |
| Treatment | 1 | 111 | 8.351 | **0.004** | 1\|month | 2.691 |
| *b) Phyto Shannon Index* |  |  |  |  | 1\|mesocosm | 0.377 |
| Treatment | 1 | 111 | 8.388 | **0.004** | 1\|month | 0.100 |
| *c) log_10_ Total Phyto Abund* |  |  |  |  | 1\|mesocosm | 0.461 |
| Treatment | 1 | 111 | 0.823 | 0.364 | 1\|month | 0.342 |
| *d) log_10_ Total Phyto Biomass* |  |  |  |  | 1\|mesocosm | 0.448 |
| Treatment | 1 | 111 | 3.934 | **0.047** | 1\|month | 0.348 |
| *e) Mean log_10_ Phyto Mass* |  |  |  |  | 1\|mesocosm | 0.861 |
| Treatment | 1 | 111 | 13.02 | **<0.001** | 1\|month | 0.353 |
| *f) Poisson log-normal (μ)* |  |  |  |  | 1\|mesocosm | 0.591 |
| Treatment | 1 | 101 | 0.061 | 0.806 | 1\|month | 0.521 |
| *g) Poisson log-normal (*σ*)* |  |  |  |  | 1\|mesocosm | 0.063 |
| Treatment | 1 | 101 | 7.532 | **<0.001** | 1\|month | 0.026 |
| *h) Prop Inedible Phyto* |  |  |  |  | 1\|mesocosm | 0.026 |
| Treatment | 1 | 111 | 6.107 | **0.013** | 1\|month | 0.045 |
| *i) log_10_ Total Zoo Biomass* |  |  |  |  | 1\|mesocosm | 0.365 |
| Treatment | 1 | 111 | 0.449 | 0.502 | 1\|month | 0.229 |
| *j) Mean log_10_ Zoo Mass* |  |  |  |  | 1\|mesocosm | 0.174 |
| Treatment | 1 | 111 | 1.442 | 0.229 | 1\|month | 0.694 |
| *k) log_10_ Cladoceran Biomass* |  |  |  |  | 1\|mesocosm | 0.273 |
| Treatment | 1 | 112 | 0.058 | 0.810 | 1\|month | 0.166 |
| *l) log_10_ Copepod Biomass* |  |  |  |  | 1\|mesocosm | 0.212 |
| Treatment | 1 | 112 | 3.136 | 0.077 | 1\|month | 0.182 |
| *m) log_10_ Daphnia Biomass* |  |  |  |  | 1\|mesocosm | 0.139 |
| Treatment | 1 | 67 | 1.592 | 0.207 | 1\|month | 0.232 |
| *n) log_10_ Eudiaptomous Biomass* |  |  |  |  | 1\|mesocosm | 0.411 |
| Treatment | 1 | 87 | 6.267 | **0.012** | 1\|month | 0.160 |
| *o) log_10_ Bosmina Biomass* |  |  |  |  | 1\|mesocosm | 0.842 |
| Treatment | 1 | 64 | 1.931 | 0.164 | 1\|month | 0.132 |
| *p) log_10_ Alona Biomass* |  |  |  |  | 1\|mesocosm | 0.577 |
| Treatment | 1 | 70 | 0.595 | 0.440 | 1\|month | 0.207 |
| *q) log_10_ Chydorus Biomass* |  |  |  |  | 1\|mesocosm | 0.336 |
| Treatment | 1 | 70 | 14.355 | **<0.001** | 1\|month | 0.117 |
| *r) log_10_ Ostracoda Biomass* |  |  |  |  | 1\|mesocosm | 0.470 |
| Treatment | 1 | 81 | 3.250 | 0.071 | 1\|month | 0.271 |
| *s) log_10_ CR* |  |  |  |  | 1\|mesocosm | 0.144 |
| Treatment | 1 | 39 | 1.981 | 0.159 | 1\|month | 0.393 |
| *t) log_10_ GPP* |  |  |  |  | 1\|mesocosm | 0.462 |
| Treatment | 1 | 44 | 4.577 | **0.032** | 1\|month | 0.202 |
| *u) log_10_ Total inorganic N* |  |  |  |  | 1\|mesocosm | 0.060 |
| Treatment | 1 | 112 | 0.297 | 0.586 | 1\|month | 0.127 |
| *v) log_10_ Phosphate* |  |  |  |  | 1\|mesocosm | 0.102 |
| Treatment | 1 | 112 | 0.875 | 0.350 | 1\|month | 0.220 |
| *w) Temperature* |  |  |  |  | 1\|mesocosm | 0.287 |
| Treatment | 1 | 4570 | 50.134 | **<0.001** | 1\|month | 4.283 |
