# Supplementary material for: Five Years of Experimental Warming Increases the Biodiversity and Productivity of Phytoplankton
Source: PLoS Biol. 2015 Dec 17;13(12):e1002324. doi: 10.1371/journal.pbio.1002324 (PMC4682994; doi:10.1371/journal.pbio.1002324)
Supplement: S2 Table — The predictors included temperature (Std Temp) as an exogenous variable (i.e., whose variance arise outside of the model) and GPP, CR, phytoplankton taxon richness (Prich), phytoplankton biomass (Pbio), and zooplankton biomass (Zbio) as endogenous variables (i.e., those whose variance the model seeks to explain). We developed a set of candidate models (see S7, S8 and S9 Figs), starting with a full model (F0), which included all feasible paths between variables. We then removed paths with the lowest p-values and compared candidate models using small sample size corrected AICc and a goodness of fit determined from D-separation tests (see Methods). Delta AICc is the difference in AICc score relative to the model with the lowest value (most parsimonious model), and Akaike Information Criterion (AIC) Weight (Wt) is the relative support for the model. Path diagrams for all candidate models are given in S7, S8 and S9 Figs. Note that goodness of fit could not be calculated from the fully saturated model (F0). The data underlying this analysis can be found in S1 Data. (DOCX) [file pbio.1002324.s014.docx]

**S2 Table. Model selection on candidate path models.**

| **Model** | **C** | **df** | **Fit (*P*)** | **AICc** | **K** | ***n*** | **Δ AICc** | **AICc Wt** |
| --- | --- | --- | --- | --- | --- | --- | --- | --- |
| F0 | - | - | - | 292.50 | 30.00 | 39 | 215.53 | 0.00 |
| F1 | 1.26 | 2 | 0.53 | 252.59 | 29.00 | 39 | 175.63 | 0.00 |
| F2 | 3.38 | 4 | 0.50 | 221.78 | 28.00 | 39 | 144.81 | 0.00 |
| F3 | 5.29 | 6 | 0.51 | 196.75 | 27.00 | 39 | 119.78 | 0.00 |
| F4 | 8.44 | 8 | 0.39 | 177.44 | 26.00 | 39 | 100.47 | 0.00 |
| F5 | 11.19 | 10 | 0.34 | 161.19 | 25.00 | 39 | 84.22 | 0.00 |
| F6 | 12.54 | 12 | 0.40 | 146.25 | 24.00 | 39 | 69.29 | 0.00 |
| F7 | 17.01 | 14 | 0.26 | 136.61 | 23.00 | 39 | 59.64 | 0.00 |
| F8 | 20.06 | 16 | 0.22 | 127.31 | 22.00 | 39 | 50.34 | 0.00 |
| F9 | 23.71 | 18 | 0.17 | 120.06 | 21.00 | 39 | 43.10 | 0.00 |
| F10 | 28.25 | 20 | 0.12 | 114.92 | 20.00 | 39 | 37.95 | 0.00 |
| **F11** | **20.24** | **12** | **0.10** | **76.97** | **16.00** | **39** | **0.00** | **1.00** |
