# Supplementary material for: Five Years of Experimental Warming Increases the Biodiversity and Productivity of Phytoplankton
Source: PLoS Biol. 2015 Dec 17;13(12):e1002324. doi: 10.1371/journal.pbio.1002324 (PMC4682994; doi:10.1371/journal.pbio.1002324)
Supplement: S3 Table — Maximum likelihood fits of the Poisson log-normal (parameters: μ and σ) model to each rank-abundance distribution along with the AIC and p-value for each parameter. The data underlying this analysis can be found in S1 Data. (DOCX) [file pbio.1002324.s015.docx]

**S3 Table. Maximum likelihood statistics for the Poisson log-normal fits**.

|  |  | **Log-normal MLE fit** | | |  |  |  |  |  |
| --- | --- | --- | --- | --- | --- | --- | --- | --- | --- |
| Pond | Treatment | | Month | Μ | | *P*-value (μ) | σ | *P*-value (σ) | AIC |
| 1 | H | | Jul-11 | 6.88 | | 2.7E-110 | 1.51 | 4.6E-12 | 422.01 |
| 1 | H | | Sept | 8.15 | | 1.5E-188 | 1.39 | 1.6E-12 | 498.97 |
| 1 | H | | Nov | 6.93 | | 8.9E-265 | 1.24 | 1.1E-18 | 672.48 |
| 1 | H | | Jan | 9.23 | | 8.7E-140 | 1.47 | 1.5E-08 | 356.88 |
| 1 | H | | March | 8.95 | | 3.7E-69 | 1.69 | 2.7E-06 | 243.76 |
| 1 | H | | May | 9.68 | | 2.3E-75 | 1.75 | 2.7E-06 | 260.46 |
| 1 | H | | Jul-12 | 7.81 | | 3.4E-110 | 1.56 | 2.6E-10 | 390.91 |
| 2 | A | | Jul-11 | 8.96 | | 1.1E-68 | 1.70 | 2.7E-06 | 243.93 |
| 2 | A | | Sept | 11.02 | | 8.0E-25 | 2.40 | 1.6E-03 | 137.15 |
| 2 | A | | Nov | 9.20 | | 7.6E-137 | 1.48 | 1.5E-08 | 356.23 |
| 2 | A | | Jan | 9.39 | | 7.1E-45 | 1.89 | 6.3E-05 | 187.05 |
| 2 | A | | March | 9.04 | | 4.5E-64 | 1.93 | 3.5E-07 | 293.01 |
| 2 | A | | May | 13.13 | | 3.0E-70 | 1.66 | 1.6E-03 | 154.57 |
| 2 | A | | Jul-12 | 11.92 | | 9.5E-30 | 2.10 | 4.7E-03 | 116.63 |
| 3 | A | | Sept | 10.71 | | 3.0E-103 | 1.79 | 3.4E-07 | 334.58 |
| 3 | A | | Nov | 11.60 | | 1.5E-76 | 1.88 | 2.2E-05 | 249.76 |
| 3 | A | | Jan | 7.80 | | 1.3E-17 | 2.23 | 6.1E-04 | 124.25 |
| 3 | A | | March | 3.70 | | 6.9E-41 | 1.05 | 1.7E-07 | 159.27 |
| 3 | A | | May | 2.20 | | 6.4E-11 | 0.98 | 5.3E-05 | 83.70 |
| 3 | A | | Jul-12 | 9.15 | | 1.4E-75 | 1.72 | 9.6E-07 | 270.79 |
| 4 | H | | Jul-11 | 10.90 | | 1.7E-79 | 1.83 | 7.7E-06 | 262.37 |
| 4 | H | | Sept | 14.31 | | 2.4E-18 | 2.84 | 1.4E-02 | 104.62 |
| 4 | H | | Nov | 9.32 | | 0.0E+00 | 1.36 | 1.2E-15 | 711.06 |
| 4 | H | | Jan | 8.42 | | 2.0E-149 | 1.52 | 3.3E-11 | 455.50 |
| 4 | H | | March | 11.25 | | 1.9E-216 | 1.34 | 1.2E-07 | 366.89 |
| 4 | H | | May | 9.98 | | 3.3E-128 | 1.55 | 1.2E-07 | 335.32 |
| 4 | H | | Jul-12 | 9.76 | | 5.8E-17 | 2.33 | 4.7E-03 | 100.19 |
| 5 | A | | Jul-11 | 12.32 | | 1.5E-15 | 2.18 | 4.5E-02 | 62.08 |
| 5 | A | | Nov | 7.63 | | 5.7E-84 | 1.67 | 2.0E-09 | 348.07 |
| 5 | A | | Jan | 9.37 | | 2.3E-74 | 1.70 | 2.7E-06 | 253.07 |
| 5 | A | | March | 9.65 | | 5.9E-82 | 1.67 | 2.7E-06 | 258.82 |
| 5 | A | | May | 14.03 | | 3.7E-11 | 3.00 | 4.6E-02 | 70.18 |
| 5 | A | | Jul-12 | 15.55 | | 1.2E-48 | 2.12 | 4.7E-03 | 145.80 |
| 6 | H | | Jul-11 | 7.49 | | 2.2E-243 | 1.23 | 1.0E-14 | 550.75 |
| 6 | H | | Sept | 9.26 | | 1.9E-186 | 1.52 | 1.2E-11 | 514.52 |
| 6 | H | | Nov | 9.66 | | 2.5E-279 | 1.38 | 5.6E-13 | 597.00 |
| 6 | H | | Jan | 4.93 | | 2.5E-42 | 1.52 | 5.1E-09 | 247.81 |
| 6 | H | | March | 7.65 | | 5.9E-96 | 1.47 | 1.6E-08 | 306.43 |
| 6 | H | | May | 6.85 | | 4.2E-108 | 1.45 | 3.4E-11 | 384.34 |
| 6 | H | | Jul-12 | 12.05 | | 0.0E+00 | 1.26 | 3.4E-15 | 853.65 |
| 7 | A | | Sept | 5.49 | | 5.0E-158 | 0.96 | 4.5E-11 | 306.15 |
| 7 | A | | Nov | 12.37 | | 2.5E-25 | 2.38 | 4.7E-03 | 121.21 |
| 7 | A | | Jan | 7.85 | | 1.1E-67 | 1.56 | 9.7E-07 | 237.21 |
| 7 | A | | March | 8.88 | | 3.6E-46 | 1.87 | 2.2E-05 | 200.53 |
| 7 | A | | Jul-12 | 10.89 | | 6.7E-52 | 2.03 | 6.3E-05 | 212.36 |
| 8 | H | | Jul-11 | 10.01 | | 2.1E-130 | 1.54 | 1.2E-07 | 336.12 |
| 8 | H | | Sept | 10.71 | | 1.0E-204 | 1.53 | 7.1E-10 | 480.87 |
| 8 | H | | Nov | 12.21 | | 0.0E+00 | 1.54 | 2.0E-13 | 763.08 |
| 8 | H | | Jan | 9.05 | | 9.4E-221 | 1.40 | 4.3E-12 | 522.83 |
| 8 | H | | March | 10.04 | | 4.7E-200 | 1.63 | 4.3E-12 | 577.45 |
| 8 | H | | May | 11.75 | | 0.0E+00 | 1.25 | 2.0E-09 | 485.83 |
| 8 | H | | Jul-12 | 14.15 | | 0.0E+00 | 1.45 | 3.3E-11 | 705.14 |
| 9 | H | | Jul-11 | 14.41 | | 5.7E-102 | 1.16 | 1.4E-02 | 99.89 |
| 9 | H | | Sept | 17.80 | | 6.2E-17 | 3.01 | 4.5E-02 | 85.29 |
| 9 | H | | Nov | 5.37 | | 4.2E-109 | 1.34 | 6.0E-15 | 442.91 |
| 9 | H | | Jan | 7.05 | | 2.2E-92 | 1.58 | 1.0E-10 | 378.90 |
| 9 | H | | March | 6.77 | | 6.2E-132 | 1.27 | 9.5E-11 | 357.84 |
| 9 | H | | May | 6.84 | | 8.4E-28 | 1.87 | 2.6E-05 | 163.89 |
| 9 | H | | Jul-12 | 8.49 | | 0.0E+00 | 1.26 | 3.8E-19 | 814.92 |
| 10 | A | | Jul-11 | 13.13 | | 7.9E-10 | 3.02 | 4.6E-02 | 66.63 |
| 10 | A | | Sept | 12.21 | | 4.4E-23 | 2.47 | 4.7E-03 | 120.25 |
| 10 | A | | Nov | 6.39 | | 2.2E-64 | 1.60 | 2.3E-09 | 302.12 |
| 10 | A | | Jan | 6.59 | | 7.1E-157 | 1.37 | 3.8E-15 | 520.03 |
| 10 | A | | March | 9.43 | | 2.9E-41 | 2.10 | 2.2E-05 | 212.69 |
| 10 | A | | May | 7.73 | | 1.2E-122 | 1.50 | 9.3E-11 | 405.60 |
| 10 | A | | Jul-12 | 10.17 | | 3.2E-165 | 1.49 | 1.5E-08 | 387.57 |
| 15 | H | | Jul-11 | 4.89 | | 1.2E-65 | 1.10 | 7.1E-08 | 196.42 |
| 15 | H | | Sept | 9.92 | | 0.0E+00 | 1.38 | 4.5E-16 | 773.65 |
| 15 | H | | Nov | 11.40 | | 0.0E+00 | 1.22 | 3.4E-15 | 811.64 |
| 15 | H | | Jan | 9.29 | | 4.7E-133 | 1.61 | 2.0E-09 | 406.49 |
| 15 | H | | March | 8.77 | | 2.3E-215 | 1.48 | 7.3E-14 | 596.85 |
| 15 | H | | May | 8.91 | | 8.0E-199 | 1.32 | 2.5E-10 | 428.31 |
| 15 | H | | Jul-12 | 10.59 | | 4.4E-287 | 1.40 | 1.2E-11 | 572.15 |
| 16 | A | | Jul-11 | 12.28 | | 9.6E-32 | 2.34 | 1.6E-03 | 149.47 |
| 16 | A | | Jan | 11.40 | | 1.9E-121 | 1.69 | 9.6E-07 | 324.25 |
| 16 | A | | March | 9.52 | | 1.3E-147 | 1.47 | 1.5E-08 | 366.32 |
| 16 | A | | May | 7.55 | | 9.6E-106 | 1.62 | 3.4E-11 | 419.93 |
| 17 | H | | Jul-11 | 7.11 | | 1.1E-60 | 1.73 | 1.7E-08 | 294.55 |
| 17 | H | | Sept | 7.67 | | 6.3E-71 | 1.67 | 4.4E-08 | 291.98 |
| 17 | H | | Nov | 9.19 | | 4.0E-282 | 1.33 | 2.0E-13 | 592.24 |
| 17 | H | | Jan | 9.74 | | 0.0E+00 | 1.34 | 2.6E-14 | 667.99 |
| 17 | H | | March | 12.04 | | 1.3E-187 | 1.60 | 4.3E-08 | 421.81 |
| 17 | H | | May | 9.42 | | 5.1E-57 | 1.96 | 2.7E-06 | 257.23 |
| 17 | H | | Jul-12 | 11.66 | | 0.0E+00 | 1.27 | 4.3E-12 | 643.20 |
| 18 | A | | Jul-11 | 6.75 | | 3.1E-44 | 1.87 | 5.7E-08 | 267.88 |
| 18 | A | | Sept | 9.26 | | 5.7E-111 | 1.60 | 4.3E-08 | 338.67 |
| 18 | A | | Nov | 8.11 | | 1.6E-128 | 1.54 | 9.2E-11 | 422.50 |
| 18 | A | | Jan | 10.96 | | 4.5E-17 | 2.61 | 4.7E-03 | 110.72 |
| 18 | A | | March | 4.95 | | 1.3E-146 | 1.13 | 1.1E-16 | 458.94 |
| 18 | A | | May | 7.36 | | 1.3E-67 | 1.75 | 6.0E-09 | 321.48 |
| 18 | A | | Jul-12 | 10.70 | | 0.0E+00 | 1.48 | 2.0E-13 | 679.38 |
| 19 | H | | Jul-11 | 7.44 | | 3.8E-215 | 1.34 | 1.3E-15 | 590.01 |
| 19 | H | | Sept | 10.63 | | 0.0E+00 | 1.28 | 2.6E-14 | 717.12 |
| 19 | H | | Nov | 10.64 | | 0.0E+00 | 1.20 | 2.2E-17 | 885.19 |
| 19 | H | | Jan | 10.26 | | 4.9E-166 | 1.58 | 2.0E-09 | 440.89 |
| 19 | H | | March | 8.89 | | 3.5E-294 | 1.33 | 9.6E-15 | 639.66 |
| 19 | H | | May | 7.96 | | 2.2E-151 | 1.49 | 4.3E-12 | 473.20 |
| 19 | H | | Jul-12 | 8.31 | | 2.4E-275 | 1.33 | 1.3E-15 | 644.90 |
| 20 | A | | Jul-11 | 10.23 | | 1.3E-143 | 1.65 | 5.5E-09 | 417.14 |
| 20 | A | | Sept | 10.61 | | 0.0E+00 | 1.47 | 1.2E-15 | 798.01 |
| 20 | A | | Nov | 9.30 | | 0.0E+00 | 1.37 | 3.5E-15 | 687.64 |
| 20 | A | | Jan | 9.48 | | 3.0E-118 | 1.69 | 5.5E-09 | 392.58 |
| 20 | A | | March | 10.86 | | 0.0E+00 | 1.32 | 2.0E-13 | 682.12 |
| 20 | A | | May | 10.47 | | 0.0E+00 | 1.42 | 2.6E-14 | 713.78 |
| 20 | A | | Jul-12 | 10.70 | | 0.0E+00 | 1.37 | 1.2E-11 | 575.67 |
