# Supplementary material for: Five Years of Experimental Warming Increases the Biodiversity and Productivity of Phytoplankton
Source: PLoS Biol. 2015 Dec 17;13(12):e1002324. doi: 10.1371/journal.pbio.1002324 (PMC4682994; doi:10.1371/journal.pbio.1002324)
Supplement: S4 Table — (DOCX) [file pbio.1002324.s016.docx]

**S4 Table. List of phytoplankton taxa observed in the warmed and ambient mesocosms.**

| **Taxon** | **Treatment** |
| --- | --- |
| *Acanthosphaera* sp. | Ambient |
| *Achnanthes* sp. | Ambient + Heated |
| *Acutodesmus* sp. | Heated |
| *Amphora* sp. | Heated |
| *Anabaena* cf. *constricta* | Ambient + Heated |
| *Anabaena* cf. *planctonica* | Heated |
| *Anabaena* sp. | Ambient + Heated |
| *Ankistrodesmus bernardii* | Ambient + Heated |
| *Ankistrodesmus falcatus* | Heated |
| *Ankistrodesmus fusiformis* | Ambient + Heated |
| *Ankyra* sp. | Heated |
| *Aphanocapsa* cf. *delicatissima* | Heated |
| *Aphanocapsa delicatissima* | Ambient |
| *Aphanocapsa holsatica* | Ambient |
| *Aphanocapsa* sp. | Ambient + Heated |
| *Aphanothece microscopica* | Ambient + Heated |
| *Aphanothece* sp. | Ambient + Heated |
| *Apiocystis brauniana* | Heated |
| *Botryococcus braunii* | Ambient + Heated |
| *Calothrix* sp. | Heated |
| *Chamaesiphon* sp. | Ambient |
| *Characiopsis lunaris* | Heated |
| *Characium* sp. | Heated |
| *Chlamydocapsa* cf. | Ambient + Heated |
| *Chlamydocapsa* sp. | Ambient + Heated |
| *Chlamydomonas* sp. | Ambient + Heated |
| *Chlorella* spp. | Ambient + Heated |
| Chlorococcales | Heated |
| *Chlorogonium* sp. | Ambient + Heated |
| *Chlorolobion* sp. | Ambient + Heated |
| *Chromulina* cf. *longiciliata* | Ambient + Heated |
| *Chromulina* sp. | Ambient + Heated |
| *Chroococcus dispersus* | Ambient |
| *Chroococcus* sp. | Ambient + Heated |
| *Chrysococcus* sp. | Ambient |
| Chrysophyte undetermined | Heated |
| *Closterium leiblenii* | Heated |
| *Closterium* spp. | Heated |
| *Closterium venus* | Heated |
| *Cocconeis placentula* | Ambient + Heated |
| *Coelosphaerium minutissimum* | Ambient |
| *Coelosphaerium* sp. | Ambient + Heated |
| *Coenocystis* sp. | Ambient + Heated |
| *Cosmarium botrytis* | Ambient + Heated |
| *Cosmarium* cf. *polygonatum* | Ambient + Heated |
| *Cosmarium* cf. *quasillus* | Heated |
| *Cosmarium humile* | Ambient + Heated |
| *Cosmarium impressulum* | Heated |
| *Cosmarium meneghinii* | Heated |
| *Cosmarium polygonatum* | Heated |
| *Cosmarium regnellii* | Ambient + Heated |
| *Cosmarium reniforme* | Ambient + Heated |
| *Cosmarium sphagnicolum* | Ambient + Heated |
| *Cosmarium* spp. | Ambient + Heated |
| *Cosmarium subcrenatum* | Ambient |
| *Cosmarium subgranatum* | Ambient + Heated |
| *Crucigeniella* sp. | Heated |
| *Cryptomonas reflexa* | Ambient + Heated |
| *Cryptomonas* spp. | Ambient + Heated |
| *Cyanogranis ferruginea* | Heated |
| *Cyanogranis* sp. | Heated |
| Cyst undetermined | Ambient + Heated |
| Cyst cf. *Goniochloris sculpta* | Ambient + Heated |
| *Desmodesmus sensu stricto* | Ambient + Heated |
| *Diatoma vulgaris* | Heated |
| *Dictyosphaerium chlorelloides* | Ambient + Heated |
| *Dictyosphaerium* sp. | Ambient + Heated |
| *Elakatothrix* sp. | Ambient + Heated |
| *Encyonopsis* sp. | Ambient |
| *Epithemia adnata* | Ambient + Heated |
| *Epithemia sorex* | Heated |
| *Euastrum verrucosum* | Ambient |
| *Eucapsis* sp. | Heated |
| *Euglena acus* | Ambient |
| *Euglena gracilis* | Ambient + Heated |
| *Euglena* sp. | Heated |
| *Euglena* spp. | Ambient + Heated |
| Flagellate undetermined | Ambient + Heated |
| Flagellate undetermined (4 flagella) | Heated |
| *Fragilaria* sp. | Ambient + Heated |
| *Geitlerinema* sp. | Ambient + Heated |
| *Geitlerinema splendidum* | Ambient + Heated |
| *Geminella* sp. | Ambient + Heated |
| *Gloeocapsopsis* sp. | Ambient + Heated |
| *Gloeocystis* cf. | Heated |
| *Gloeocystis* sp. | Heated |
| *Golenkinia* cf. | Heated |
| *Gomphonema* spp. | Ambient + Heated |
| *Goniochloris sculpta* | Ambient + Heated |
| *Gonium* cf. | Ambient |
| *Granulocystis* sp. | Ambient |
| *Gymnodinium* sp. | Ambient + Heated |
| Isolated cell undetermined | Ambient + Heated |
| *Kirchneriella* sp. | Heated |
| *Koliella* sp. | Heated |
| *Lepocinclis* sp. | Heated |
| *Lobomonas* sp. | Ambient |
| *Mallomonas* sp. | Ambient |
| *Merismopedia* cf. *insignis* | Ambient + Heated |
| *Merismopedia* sp. | Heated |
| *Merismopedia tenuissima* | Heated |
| *Microcystis* cf. *natans* | Ambient + Heated |
| *Monoraphidium arcuatum* | Ambient + Heated |
| *Monoraphidium circinale* | Ambient + Heated |
| *Monoraphidium contortum* | Ambient + Heated |
| *Monoraphidium griffithii* | Ambient + Heated |
| *Monoraphidium komarkovae* | Ambient + Heated |
| *Monoraphidium minutum* | Ambient + Heated |
| *Navicula* spp. | Ambient + Heated |
| *Nephrocytium aghardianum* | Ambient + Heated |
| *Nephrocytium limneticum* | Heated |
| *Nephrocytium* sp. | Heated |
| *Nephrodiella* sp. | Ambient |
| *Nephroselmis* sp. | Ambient |
| *Nitzschia* spp. | Ambient + Heated |
| Nostocales | Ambient + Heated |
| *Oedogonium* sp. | Ambient + Heated |
| *Oocystis + Granulocystis* | Heated |
| *Oocystis* spp. | Ambient + Heated |
| *Oscillatoria limosa* | Ambient + Heated |
| *Oscillatoria* sp. | Ambient |
| Oscillatoriales | Ambient + Heated |
| *Pandorina* sp. | Heated |
| *Paulschulzia* sp. | Ambient + Heated |
| *Pediastrum boryanum* | Ambient + Heated |
| *Pediastrum tetras* | Ambient + Heated |
| Pennate diatom | Ambient + Heated |
| *Peridinium willei* | Ambient + Heated |
| *Petalomonas* sp. | Ambient + Heated |
| *Phacus acuminatus* | Heated |
| *Phacus* spp. | Heated |
| *Phacus tortus* | Ambient |
| *Phormidium* sp. | Ambient + Heated |
| *Plagioselmis nannoplanctica* | Ambient + Heated |
| *Planktolyngbya limnetica* | Heated |
| *Pleurotaenium trabecula* | Ambient + Heated |
| *Pseudanabaena catenata* | Ambient + Heated |
| *Pseudanabaena* cf. *moniliformis* | Heated |
| *Pseudanabaena dictyothalla* | Ambient |
| *Pseudanabaena limnetica* | Ambient + Heated |
| *Pseudanabaena mucicola* | Ambient + Heated |
| *Pseudanabaena* sp. | Ambient + Heated |
| *Rhodomonas* sp. | Ambient + Heated |
| *Scenedesmus* cf. *pseudodenticulatus* | Ambient + Heated |
| *Scenedesmus* gr. *Abundantes Spinosi* | Ambient + Heated |
| *Scenedesmus* gr. *Armati* | Ambient + Heated |
| *Scenedesmus* gr. *sensu stricto* | Ambient + Heated |
| *Scenedesmus magnus* | Heated |
| *Snowella* sp*.* | Ambient + Heated |
| *Spermatozopsis exsultans* | Heated |
| *Sphaerellopsis* sp. | Ambient |
| *Sphaerocystis* cf. | Heated |
| *Sphaerocystis* sp. | Ambient + Heated |
| *Spirogyra* sp. | Heated |
| *Staurastrum striolatum* | Ambient + Heated |
| *Synechococcus* sp. | Ambient + Heated |
| *Synechocystis* cf. | Heated |
| *Tetraedron caudatum* | Ambient + Heated |
| *Tetraedron minimum* | Ambient + Heated |
| *Tetraedron triangulare* | Ambient + Heated |
| *Tetraselmis* sp. | Ambient |
| *Tetrastrum* sp. | Ambient + Heated |
| *Tetrastrum triangulare* | Heated |
| *Trachelomonas abrupta* | Ambient + Heated |
| *Trachelomonas hispida* | Ambient + Heated |
| *Trachelomonas* sp. | Ambient + Heated |
| *Trachelomonas* spp. | Ambient + Heated |
| *Trachelomonas volvocina* | Ambient + Heated |
| Undetermined | Ambient + Heated |
| Volvocales | Ambient |
| *Willea villhelmii* | Ambient + Heated |
