# Supplementary material for: Five Years of Experimental Warming Increases the Biodiversity and Productivity of Phytoplankton
Source: PLoS Biol. 2015 Dec 17;13(12):e1002324. doi: 10.1371/journal.pbio.1002324 (PMC4682994; doi:10.1371/journal.pbio.1002324)
Supplement: S5 Table — (DOCX) [file pbio.1002324.s017.docx]

**S5 Table. List of zooplankton taxa observed in the warmed and ambient mesocosms.**

| **Taxon** | **Treatment** |
| --- | --- |
| *Alona* spp. | Ambient |
| Arachnoidea | Heated |
| *Bosmina* spp. | Ambient + Heated |
| Calanoida unid | Ambient + Heated |
| *Centropyxis* spp. | Ambient + Heated |
| Ceratopogonidae larvae | Ambient |
| *Ceriodaphnia* spp. | Ambient + Heated |
| Chironomidae larvae | Ambient + Heated |
| *Chydorus* spp. | Ambient + Heated |
| Ciliata unid | Ambient + Heated |
| Cladocera unid | Ambient + Heated |
| Coleoptera larvae | Ambient + Heated |
| Copepoda unid | Ambient + Heated |
| Culicidae | Heated |
| Cyclopoida | Ambient + Heated |
| *Daphnia* spp. | Ambient + Heated |
| Diptera larvae | Ambient + Heated |
| *Diaptomus* spp. | Ambient + Heated |
| *Filinia* spp. | Ambient + Heated |
| Flagellata | Ambient + Heated |
| Gastropoda | Ambient + Heated |
| Harpacticoida | Heated |
| Hydracarina | Ambient + Heated |
| Insecta larvae | Ambient + Heated |
| *Keratella* spp. | Ambient + Heated |
| *Lecane* spp. | Ambient + Heated |
| *Mytilina* | Heated |
| Nematoda | Ambient + Heated |
| Odonata larvae | Heated |
| Oligochaeta | Ambient + Heated |
| Ostracoda | Ambient + Heated |
| Plecoptera | Ambient |
| Polychaeta larvae | Ambient |
| *Rotatoria* sp. | Ambient + Heated |
| Rotifera | Ambient + Heated |
| *Synchaeta* spp. | Ambient + Heated |
| *Trichocerca* spp. | Heated |
| Trichoptera larvae | Heated |
| Turbellaria | Ambient + Heated |
