# Supplementary material for: Five Years of Experimental Warming Increases the Biodiversity and Productivity of Phytoplankton
Source: PLoS Biol. 2015 Dec 17;13(12):e1002324. doi: 10.1371/journal.pbio.1002324 (PMC4682994; doi:10.1371/journal.pbio.1002324)
Supplement: S6 Table — (DOCX) [file pbio.1002324.s018.docx]

**S6 Table. Average annual concentrations of total inorganic Nitrogen and orthophosphate.**

| **Pond** | **Treatment** | **[Total inorg. N] (μmol L^-1^)** | **[PO_4_^3-^] (μmol L^-1^)** |
| --- | --- | --- | --- |
| 1 | Heated | 12.38 | 0.29 |
| 2 | Ambient | 12.12 | 0.38 |
| 3 | Ambient | 25.44 | 0.51 |
| 4 | Heated | 19.50 | 0.31 |
| 5 | Ambient | 10.47 | 0.31 |
| 6 | Heated | 15.43 | 0.25 |
| 7 | Ambient | 26.47 | 0.19 |
| 8 | Heated | 18.05 | 0.17 |
| 9 | Heated | 14.64 | 0.22 |
| 10 | Ambient | 11.50 | 0.16 |
| 15 | Heated | 16.61 | 0.22 |
| 16 | Ambient | 15.74 | 0.24 |
| 17 | Heated | 20.72 | 0.17 |
| 18 | Ambient | 13.63 | 0.21 |
| 19 | Heated | 20.14 | 0.17 |
| 20 | Ambient | 18.02 | 0.23 |
